# Natural vs. Synthetic Phosphate as Efficient Heterogeneous Compounds for Synthesis of Quinoxalines

**DOI:** 10.3390/ijms222413665

**Published:** 2021-12-20

**Authors:** Abbas Amini, Azadeh Fallah, Ahmad Sedaghat, Ahmad Gholami, Chun Cheng, Anju R. Gupta

**Affiliations:** 1 Centre for Infrastructure of Engineering, Bld Z, Locked Bag 1797, Kingswood Campus, Western Sydney University, Penrith, NSW 2751, Australia; 2 Department of Mechanical Engineering, Australian College of Kuwait, Mishref, Safat 13015, Kuwait; a.sedaghat@ack.edu.kw; 3 Department of Chemistry, Payame Noor University, Tehran 19395-4697, Iran; azadehfallah84@gmail.com; 4 Pharmaceutical Sciences Research Center, Shiraz University of Medical Sciences, Shiraz 71348-14336, Iran; Gholami@sums.ac.ir; 5 Department of Pharmaceutical Biotechnology, School of Pharmacy, Shiraz University of Medical Sciences, Shiraz 71348-14336, Iran; 6 Department of Materials Science and Engineering, Southern University of Science and Technology (SUSTech), Shenzhen 518055, China; chengc@sustech.edu.cn; 7 Department of Mechanical Engineering, Industrial and Manufacturing Engineering, The University of Toledo, Toledo, OH 43606, USA; anju.gupta@utoledo.edu

**Keywords:** heterogeneous catalyst, natural phosphate, fluorapatite, quinoxalines, apatite, nano-bio-chemistry

## Abstract

Natural phosphate (NP) and synthetic fluorapatite phosphate (SFAP) were proposed as stable, inexpensive, readily available and recyclable catalysts for the condensation of 1,2-diamines with 1,2-dicarbonyls in methanol to afford quinoxaline at room temperature. NP provided as high as 92–99% yield for quinoxalines in short reaction times (i.e., 1–45 min), while SFAP created quinoxalines with 87–97% yield in 60–120 min. From the chemical analyses, X-ray fluoresecency, X-ray diffraction, energy dispersive X-ray and Fourier-transform infrared spectroscopy methods, two main phases (CaO, P_2_O_5_) appeared in NP together with other low content phases (SiO_2_, Fe_2_O_3_). Compared to other phases, apatite (CaO and P_2_O_5_ as Ca10(PO_4_)_6_) played a major role in the catalytic activity of NP. SFAP with similar Ca/P atomic ratio showed a relatively lower catalytic activity than NP for the condensation of 1,2-diamine with 1,2-dicarbonyl in methanol at ambient temperature. To investigate the recyclability of catalysts, the surface properties of NP and 6-recycled NP were investigated using scanning electron microscopy, energy dispersive X-ray and Brunauer–Emmett–Teller and Barrett–Joyner–Halenda methods. Some differences were observed in NP and 6-recycled NP’s particle size, surface area, the volume and size of pores, and the content of elements; nevertheless, the use–reuse process did not noticeably change the catalytic property of NP.

## 1. Introduction

Nature-based benign procedures require mild reaction conditions, easy purification, waste management and kinetic enhancement [1]. It becomes clear that the association of heterogeneous catalysis presents an open window of opportunities to advance chemical reactions because of the potential for enhancing the efficiency, selectivity and reusability of catalysts. Natural agents and biodegradable heterogeneous catalysts, i.e., inorganic solids (zeolite [2], silica [3], alumina [4], clay [5], etc.), can play a key role in the field of sustainable chemistry by reducing cost and waste disposal through mild catalytic processes [6]. In parallel, mineralogical classes of natural phosphate (NP), which generally belong to the family of phosphocalcic apatites (Ca_10_(PO_4_)_6_X_2_, X=OH and F), can be exploited to acquire hydroxyl and fluorapatite in their pure state [7,8,9]. Natural phosphate (apatite) and synthetic fluorapatite phosphate have been used [8,10] as the catalysts for Claisen–Schmidt condensation [11], the hydration of nitriles [12] and the oxidation of cyclic ketones to keto acids [13], tetraketones [14], xanthenes [14,15] and 1,4-disubstituted-1,2,3-triazoles [15]. In the present study, the main components of the newly used NP are investigated and its catalytic effects for the synthesis of quinoxaline are assayed.

Quinoxalines have attracted significant attention due to their various applications as base materials for dyes [16], optoelectronic and luminescent materials [17], semiconductors [18] and inhibitors [19]. Quinoxaline moiety has been used for the synthesis of biologically active compounds with antitumor [20,21], antiviral [22], anti-inflammatory [23], anti-HIV [24] and anticancer [25] properties. Various strategies for the synthesis of quinoxalines have been proposed by using different catalysts, such as aqueous hydrofluoric acid and gadolinium chloride [26], magnetic clayzic [27], alkyl sulfonate functionalized metal organic framework (MOF), MIL-101-Cr–NH–RSO_3_H [28], thiamine hydrochloride (VB_1_) [29], Zr-CAP-SG [30], Cu (BDC) [31], sulfonated nanoclay [32], arabic gum [1], sulphated polyborate [33], nano-particles ZrO_2_ [34], nanostructured pyrophosphate [21] and magnetic material separated from coal fly ash [35] through the condensation of 1,2-diketones with 1,2-diamines. These methods need a higher quantity of catalyst and suffer from a long reaction time, harsh conditions, multi-step processes for the preparation of the catalyst and high costs.

**Scheme 1:**
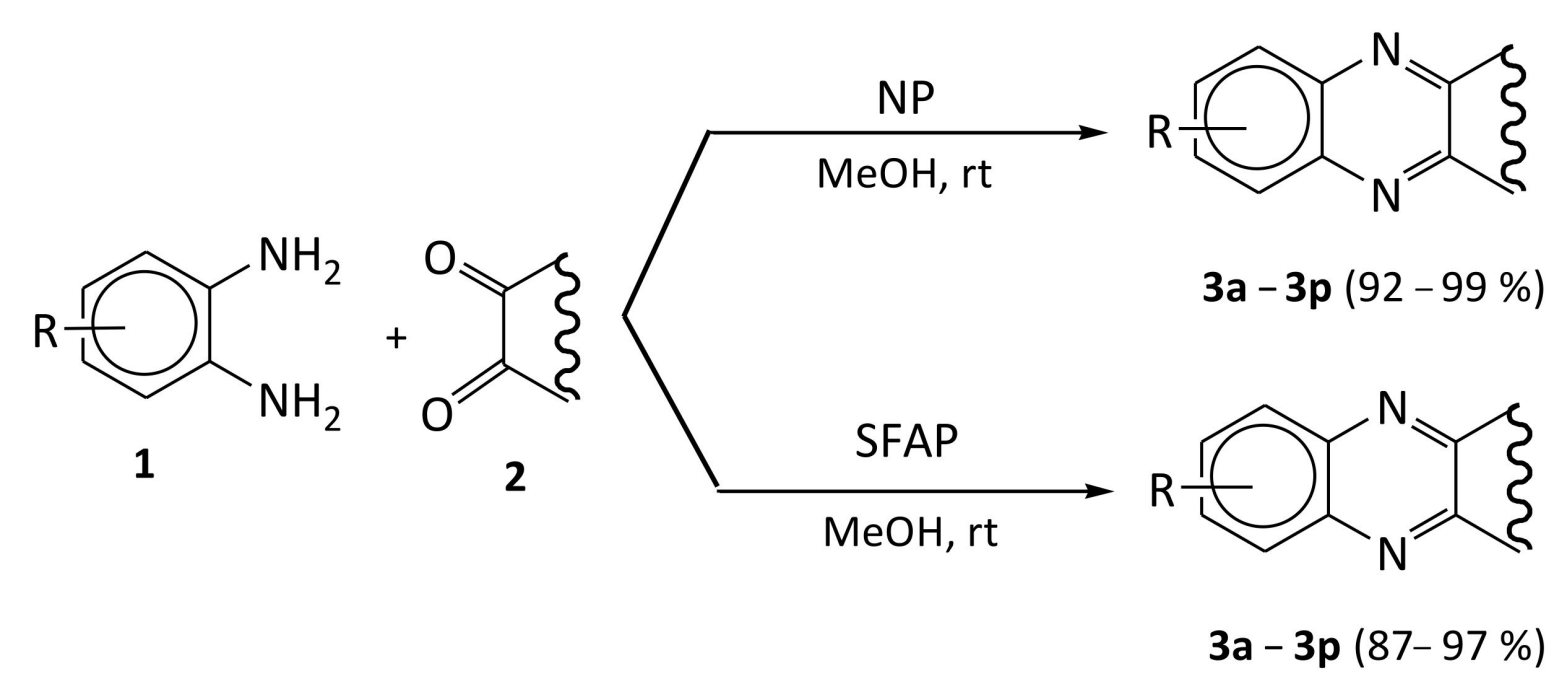
NP and SFAP as heterogeneous catalysts for the synthesis of quinoxalines.

Here, NP and SFAP were used as the catalysts (see Scheme 1) for the synthesis of quinoxaline with excellent yield under mild conditions. As the structure of NP was similar to that of fluorapatite (Ca_10_(PO_4_)_6_F_2_), we prepared SFAP through the co-precipitation method [36] and compared its structure and catalytic activity with NP. In short, NP was found as a perfect natural catalyst and performed better than SFAP in the condensation reaction of a series of 1,2-diketones with 1,2-diamines in methanol to successfully afford quinoxalines with excellent yields at room temperature (rt).

## 2. Results and Discussion

### 2.1. Characterization of NP and SFAP

The structures of NP and SFAP were characterized using Fourier-transform infrared spectroscopy (FT-IR), scanning electron microscopy (SEM), energy dispersive X-ray analysis (EDX), Brunauer–Emmett–Teller and Barrett–Joyner–Halenda (BET and BJH) methods, X-ray fluorescence (XRF) and X-ray diffraction (XRD). The NP structure was similar to the reported synthetic fluorapatite in the literature [36] and Morocco’s natural phosphate [37,38]. The structure of SFAP was found to be similar to the reported synthetic fluorapatite in the literature [36]. These analyses could help us to assess the properties of these inorganic compounds and determine the rationale for their catalytic activities and their differences.

#### 2.1.1. FT-IR Spectroscopy

**Figure 1:**
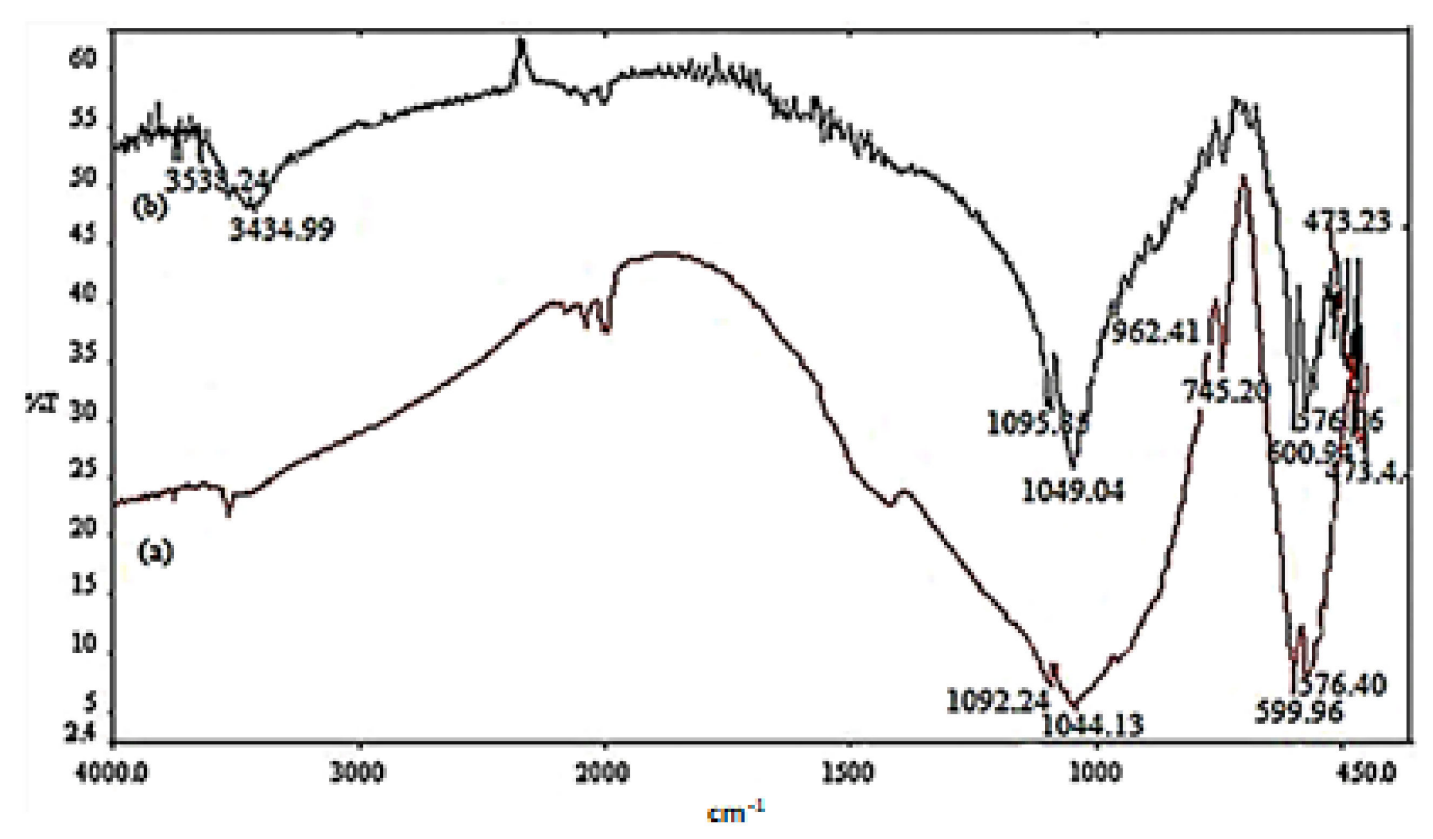
FT-IR spectra of: (**a**) NP, and (**b**) SFAP.

The FT-IR spectra of NP and SFAP are presented in Figure 1. A number of bands in the spectra were located between 1000–1100 cm^−1^, including those at 1092-5 and 1044-9 cm^−1^. These bands were assigned to triply degenerate asymmetric stretching modes of P―O (ν_3_) vibrations of the phosphate group. The peak at 962 cm^−1^ was assigned to a non-degenerated symmetric stretching mode (ν_1_) of P―O bond of the phosphate group. The bands at 600 and 576 cm^−1^ were from triply degenerate asymmetric bending modes (ν_4_) of phosphate O―P―O bonds, while the weak peaks at 473 cm^−1^ were ascribed to doubly degenerate bending mode (ν_2_) of the phosphate group (O―P―O bond) [39,40,41,42]. 

#### 2.1.2. XRF Analysis 

The chemical compositions of NP were determined as P_2_O_5_ (37.61 %), CaO (48.53 %), MgO (1.16 %),SiO_2_ (4.26 %), Fe_2_O_3_ (5.26 %), F (2.86 %), Cl (0.13 %), TiO_2_ (0.25 %), Ce_2_O_3_ (0.86 %), La_2_O_3_ (0.4 %) Nd_2_O_3_ (0.42 %), Y_2_O_3_ (0.26 %), Al_2_O_3_ (0.19 %), MnO (0.08 %), Na_2_O (0.28 %), K_2_O (0.06 %) SO_3_ (0.32 %) [14], and for SFAP, they were: P_2_O_5_ (40.71 %) and CaO (57.69 %) and F (2.16 %). The chemical components of NP were similar to SFAP; this is also supported by the data found in the literature on fluorapatite (Ca_10_(PO_4_)_6_F_2_) in Morocco natural phosphate [43]. According to previous reports, the Ca/P atomic ratio of apatites is approximately 1.67, based on their chemical formula for standard apatite; the calcium deficiency can lower the Ca/P atomic ratio to 1.5 [42,44,45]. The Ca/P atomic ratios of NP (48.5/37.6) and SFAP (57.69/40.71) were reported with the values of 1.61 and 1.58, respectively, which are within the acceptable range for apatite (1.5–1.67). 

#### 2.1.3. XRD Analysis

**Figure 2:**
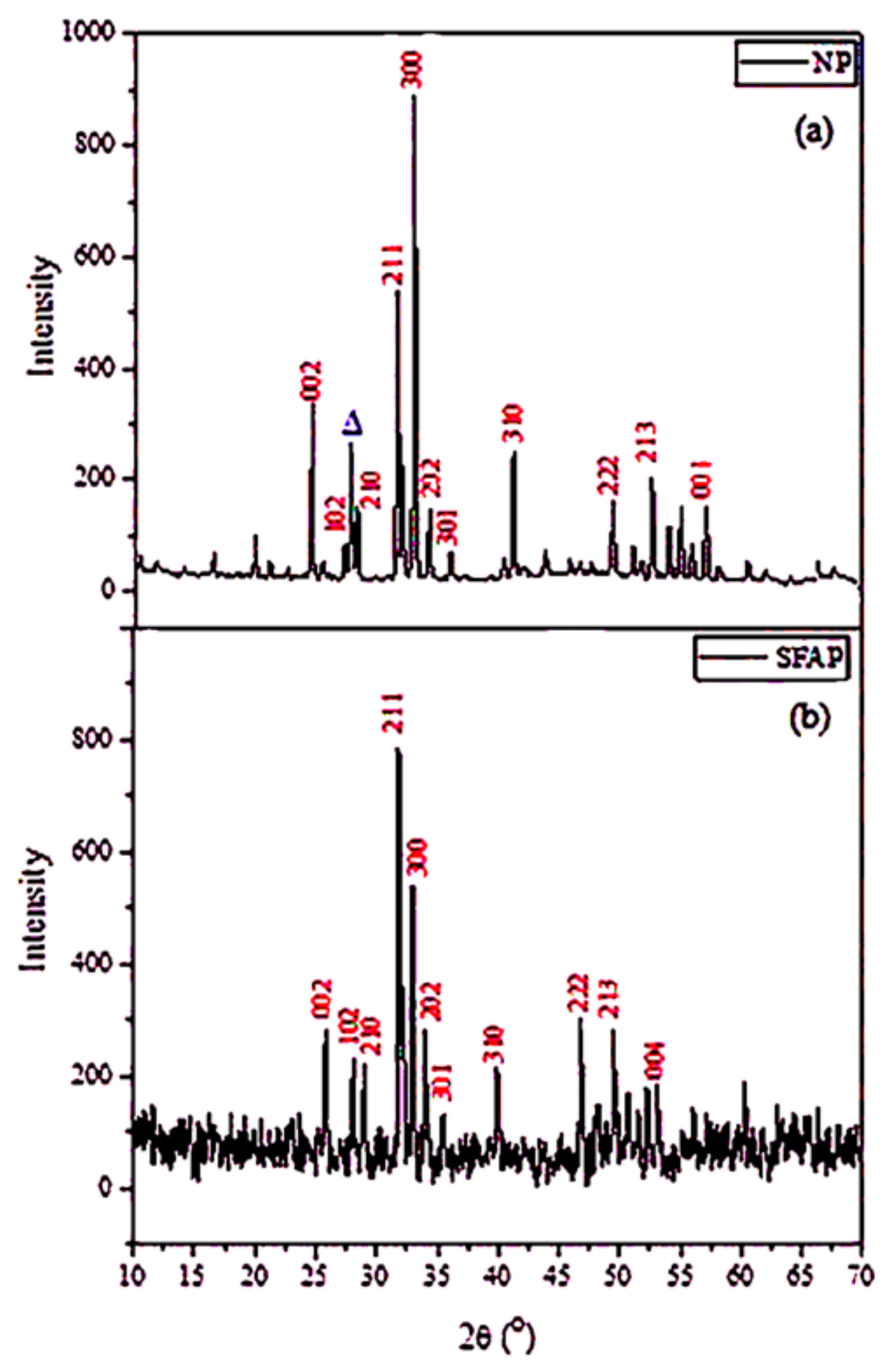
XRD patterns of: (**a**) NP; the blue triangle is showing the diffraction peak of coesite, SiO_2_ (JCPDS 72-1601) [48], and (**b**) SFAP. (Red numbers represent the crystalline planes).

XRD patterns of NP [14] and SFAP (Figure 2) show that they are well-crystalline with fluorapatite-type structures and the peaks fitted with PDF#01-015-876 [46] (Figure 1b, Supplementary Information, SI); the sharp and distinct peaks demonstrate a high degree of crystallinity. Based on the available crystallographic data for NP and SFAP, NP belongs to the same space group as SFAP; the hexagonal system of crystallite with the space group of P6_3_/m. The lattice parameters of NP and SFAP are in excellent agreement with the standard data [36]; for NP, a = 9.381 Å and c = 6.900 Å and V = 526.86 Å^3^, and for SFAP, a = 9.3684 Å and c = 6.8841 Å and V = 523.25 Å^3^. The crystallite sizes of NP and SFAP were calculated through the Debye–Scherrer equation (Equation (1)) using XRD data of NP and SFAP and X’Pert High Score Plus program:

*D = 0.9λ(β_0.5_)*^−1^*(cos θ)*^−1^ (1)

where *D* is the average crystallite size (156 nm for NP and 76 nm for SFAP); *λ*, the wavelength of X-ray used (0.154 nm for Cu); *β_0.5_*, the line broadening at the half maximum intensity after subtracting the instrumental line broadening in radians; and *θ*, Bragg angle in degree [47]. The average values of crystallite size determined for both NP and SFAP particles show that, for (0 0 2), (2 1 1), (3 0 0), (3 1 0) Miller’s planes (except (2 2 2)), they have higher intensity than others (see Figure 2). Despite the larger average crystallite size of NP than SFAP (Table 1), NP possesses higher catalytic activity in the synthesis of quinoxaline.

#### 2.1.4. pH Value of Surface 

**Figure 3:**
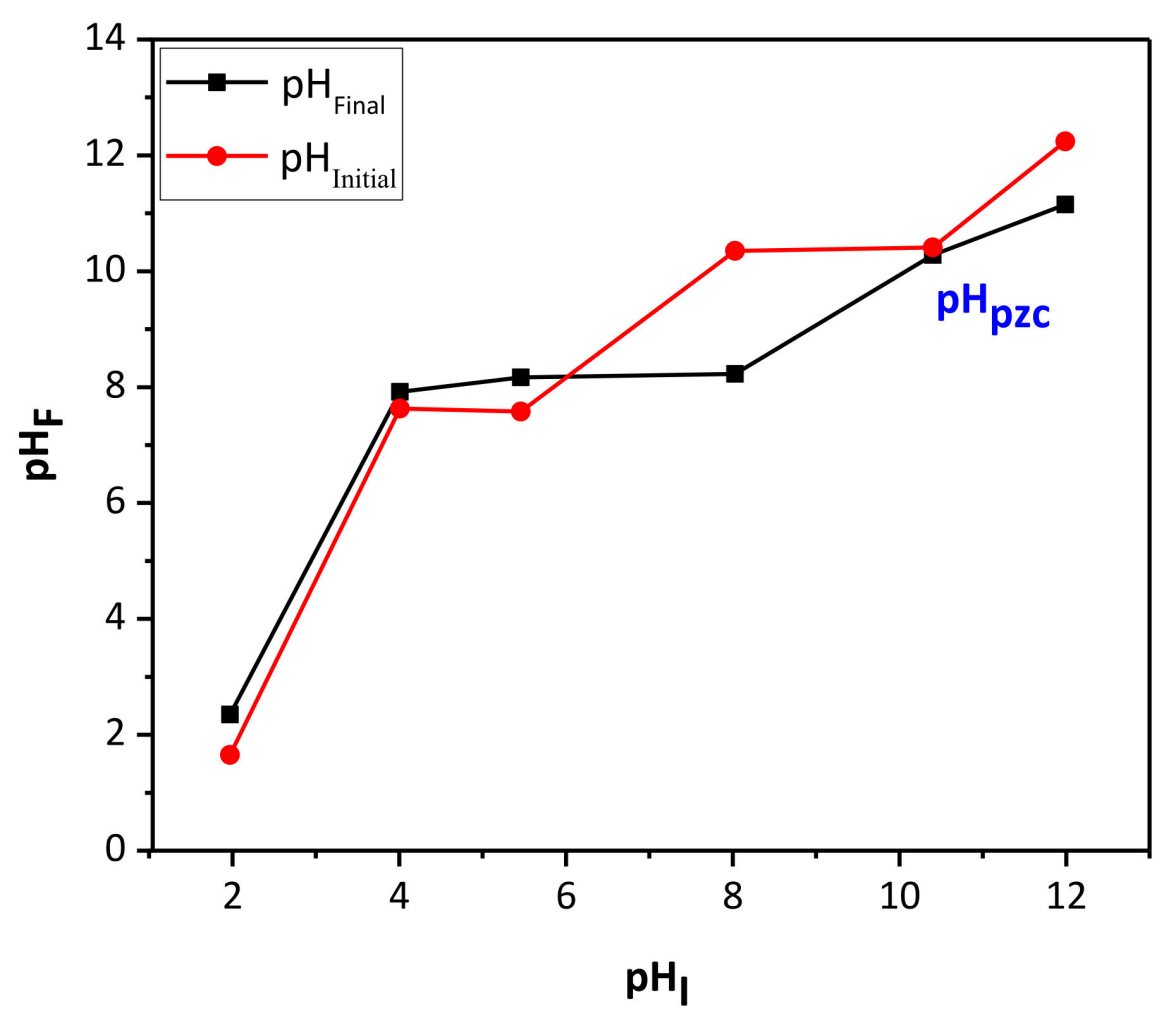
Determining pH_pzc_ of NP.

To obtain the basicity of the surface of NP, the pH of the surface was determined as 10.34 at the point of zero charge (pH_pzc_) (Figure 3) [49]. Thus, the presence of basic sites on the surface of NP induced the co-adsorption of substrate molecules. This alteration plays a key role in devising a more effective approach on the collision of the catalyst with the organic substrate. The pH_pzc_ for a mineral is the value at which the surface has a negative charge, so, when pH < pH_pzc,_ the surface possesses positive charge in a solution, whereas the negative charge occurs when pH > pH_pzc_. These effects can be explained by the deprotonation or protonation of phosphate P–O(H) [50] and calcium oxide Ca–O(H) [51] groups in the framework of NP. So, it is reasonable to choose the external surface of NP as the basic surface.

### 2.2. Reusability of Catalyst

#### 2.2.1. BET and BJH Methods

**Figure 4:**
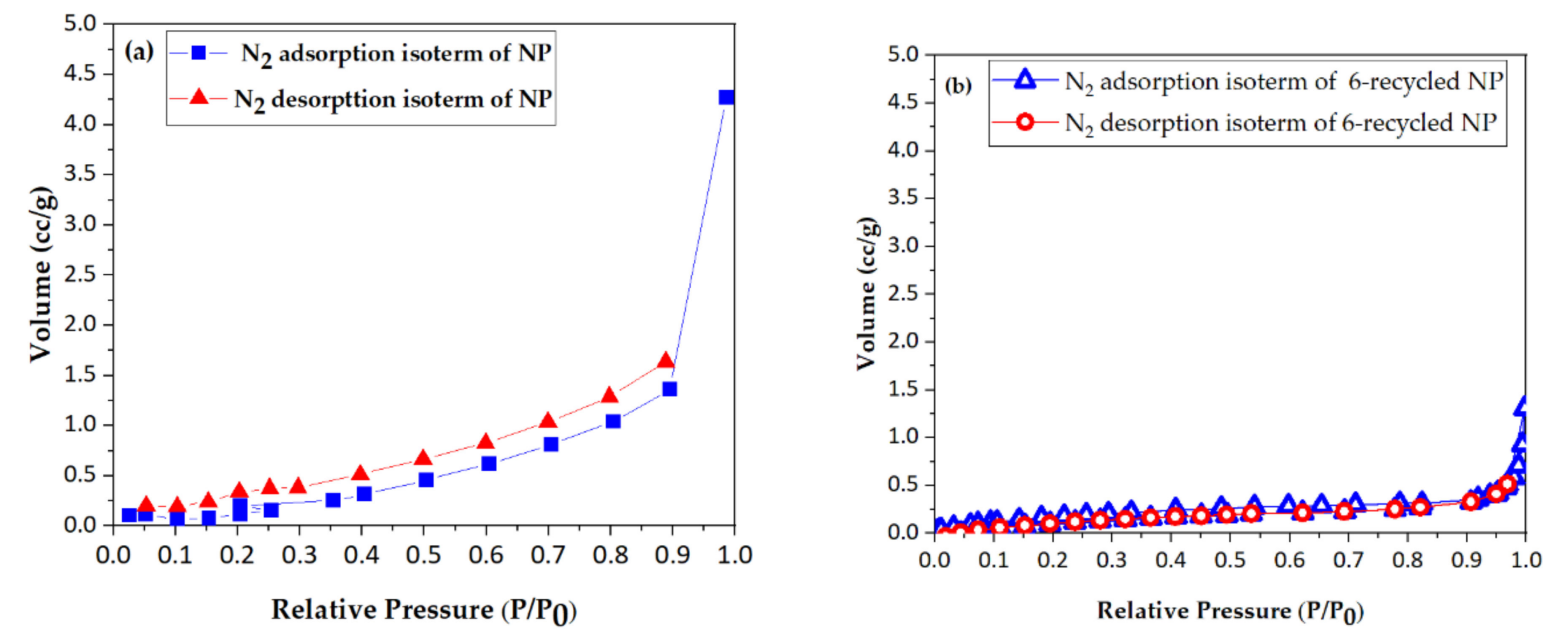
N_2_ adsorption–desorption isotherm (BET) of: (**a**) NP, and (**b**) 6-recycled NP.

Recent studies suggest that finding the specific surface area is often a prerequisite for quantizing and interpreting adsorption properties [15]. To determine the role of NP catalytic activity in the model reaction, we characterized the specific surface areas of NP after six recycling runs (6-recycled NP) for the model reaction. The specific surface areas and pore size distributions of NP and 6-recycled NP were measured through the nitrogen adsorption–desorption isotherms using BET and BJH methods. According to BET results, the adsorption isotherms of NP and 6-recycled NP were classified as type IV with a H_3_ hysteresis loop at P/P_0_ ∼ 0.98; then, according to Brunauer–Deming–Deming–Teller (BDDT) classification [52], they were categorized as mesoporous solids [53] (Figure 4). 

**Figure 5:**
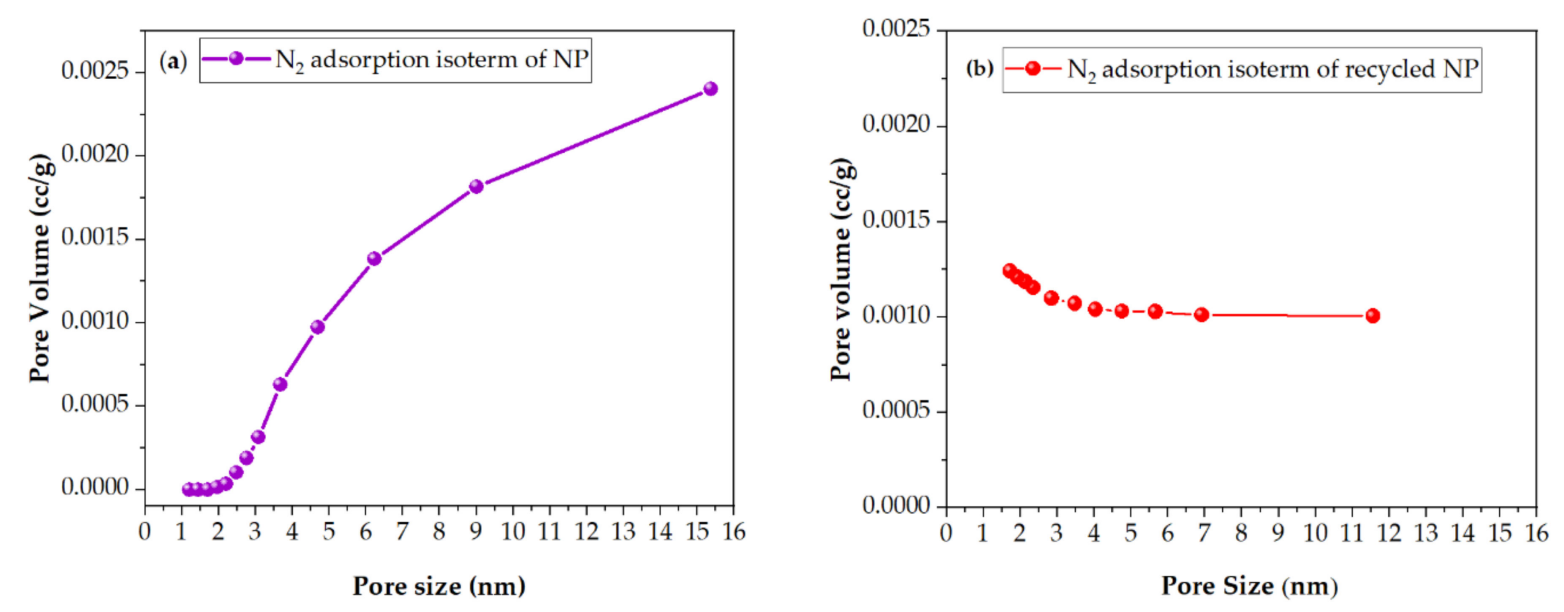
Pore size distributions curve (BJH) of: (**a**) NP, and (**b**) 6-recycled NP.

Pore volume and size distributions of NP and 6-recycled NP were analyzed by using the BJH method for the N_2_ adsorption branch isotherms (Figure 5, Table 2). The surface areas of these particles are considered low [54] as per S_BET_ = 2.081 and S_BJH_ = 1.948 m^2^g^−1^ for NP, and S_BJH_ = 0.761 and S_BJH_ = 0.426 m^2^g^−1^ for recycled NP. There is no noticeable porosity as per the pore volume of 0.007 cm^3^g^−1^ for NP with the average size of 3.675 nm, and 0.001 cm^3^g^−1^ for 6-recycled NP with the average pore size of 5.784 nm. These results are in good agreement with the reported data for Morocco natural phosphate [12,55]. By comparing the specific surface area and average pore volume of NP and 6-recycled NP, it is concluded that the recycled NP has lower porosity and specific surface area, which can be responsible for its lower catalytic activity [56]. The very low surface area of 6-recycled NP may facilitate the strong adsorption of a small amount of either quinoxaline or other unknown by-products [54]. The reduction in surface area in recycled NP may also occur during the calcination, attributing to the coalescence of NP particles [57]. 

#### 2.2.2. SEM Analysis

**Figure 6:**
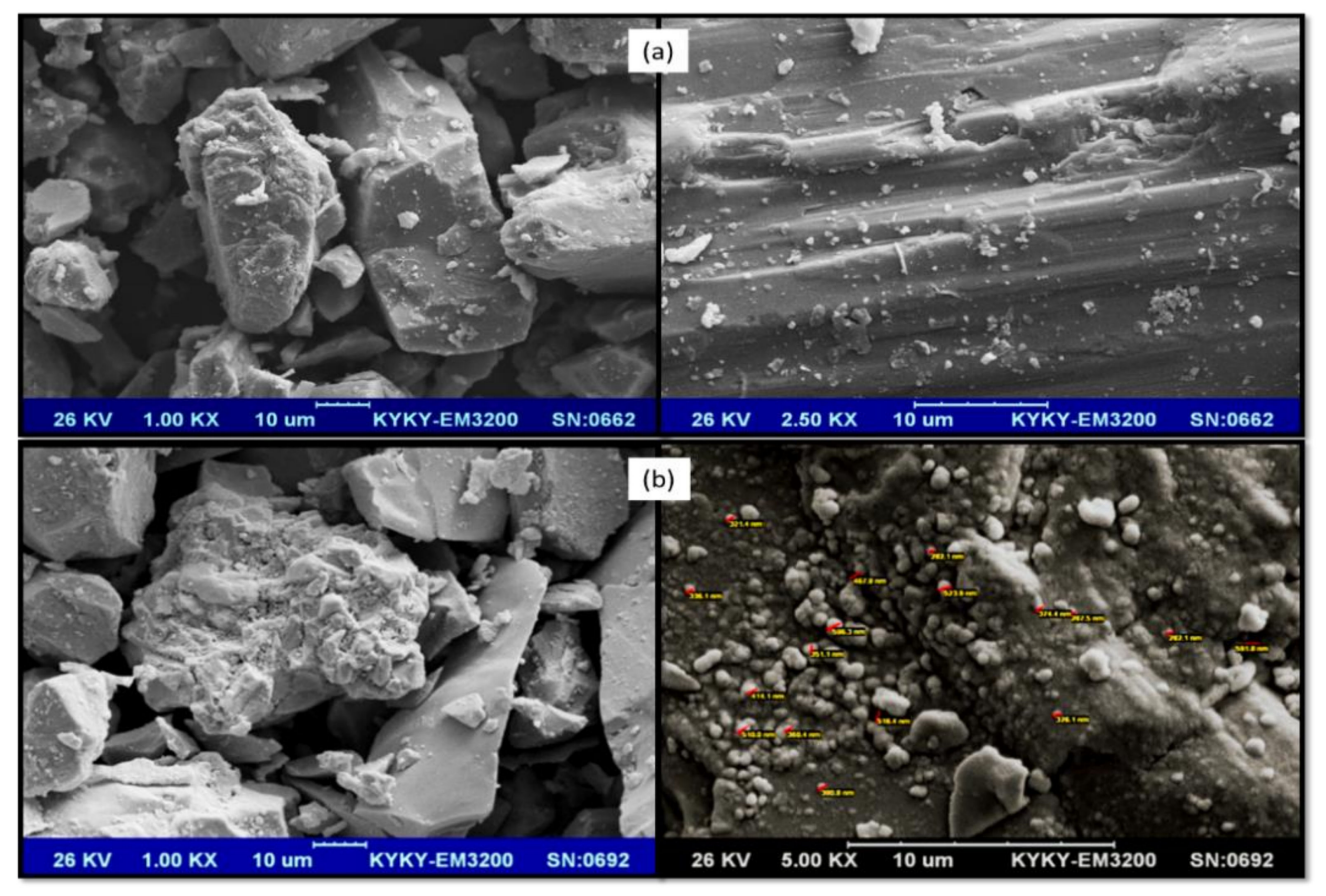
SEM images of: (**a**) NP, and (**b**) 6-recycled NP.

The surface morphology of NP and 6-recycled NP was investigated using the SEM method (Figure 6). As depicted in Figure 6b, the 6-recycled NP is shown at different magnifications to visualize the detailed surface change which indicates insignificant differences. It contains particles with an average diameter of 282–591.8 nm and a uniform distribution without significant aggregation [53]. The small particles in Figure 6b may be hematite (Fe_2_O_3_) which are replaced with hydroxyl ions from phosphate and have catalytic activity [58].

#### 2.2.3. EDX Analysis

**Scheme 2:**
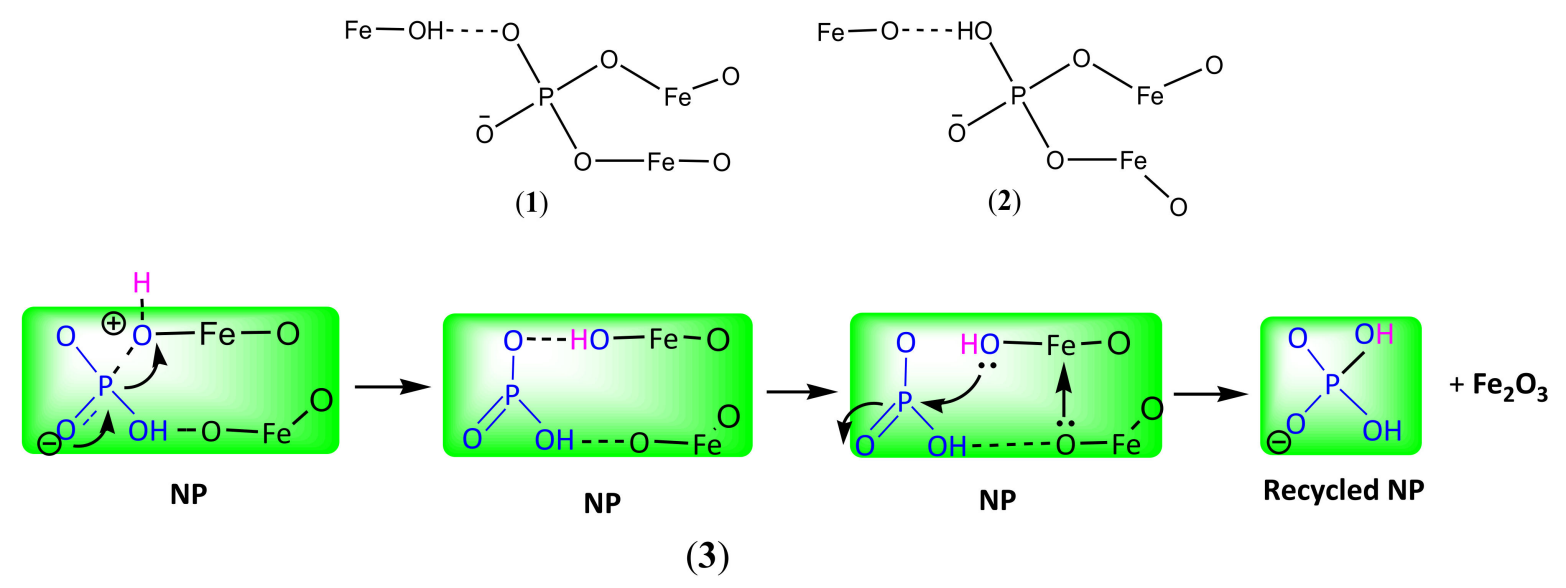
NP is composed of different oxides (P_2_O_5_, CaO, Fe_2_O_3_ and SiO_2_) that are placed together in its crystalline structure. In the proposed mechanism for the reaction by NP as the catalyst (Scheme 3), surface oxygens of NP adsorbed hydrogens of amino group (N^_^H) through hydrogen bonding to form hydroxyl group on these oxides like hematite (Fe_2_O_3_). Through hydrogen bonding, this protonated hematite is adsorbed on phosphate to form binuclear complex of hematite (Fe_2_O_3_) and phosphate (P_2_O_5_, PO_4_^3-^), or HFe_3_PO_7_ (**1** and **2**). Hydrogen atom in hydrogen bonding could be closer either to oxygen atoms of (1) hematite or (2) phosphate. (3) Proposed mechanism for the Fe_2_O_3_ losses from NP during the reactions [63].

**Scheme 3:**
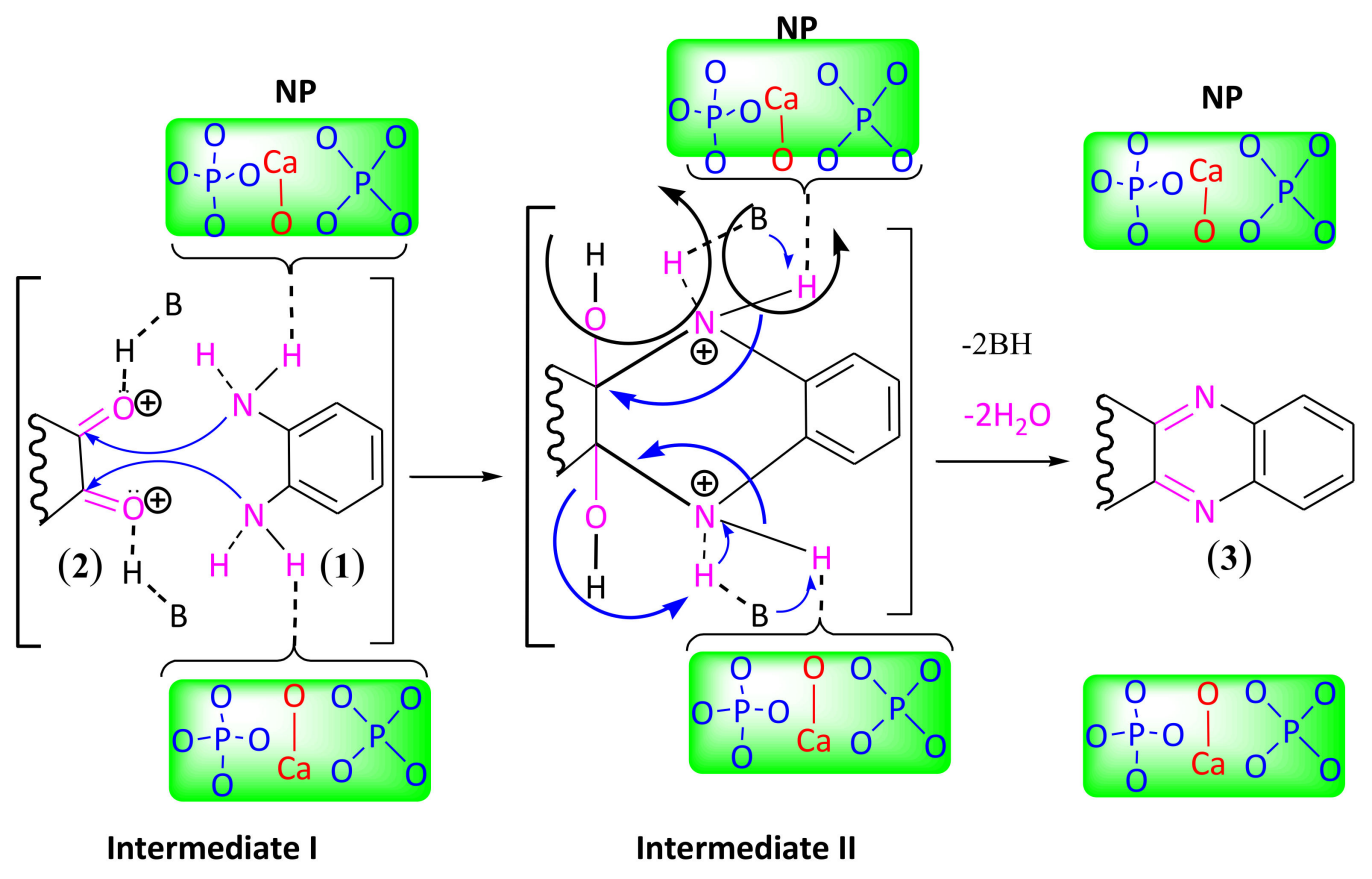
Proposed mechanism for the synthesis of quinoxaline using NP as the catalyst.

**Figure 7:**
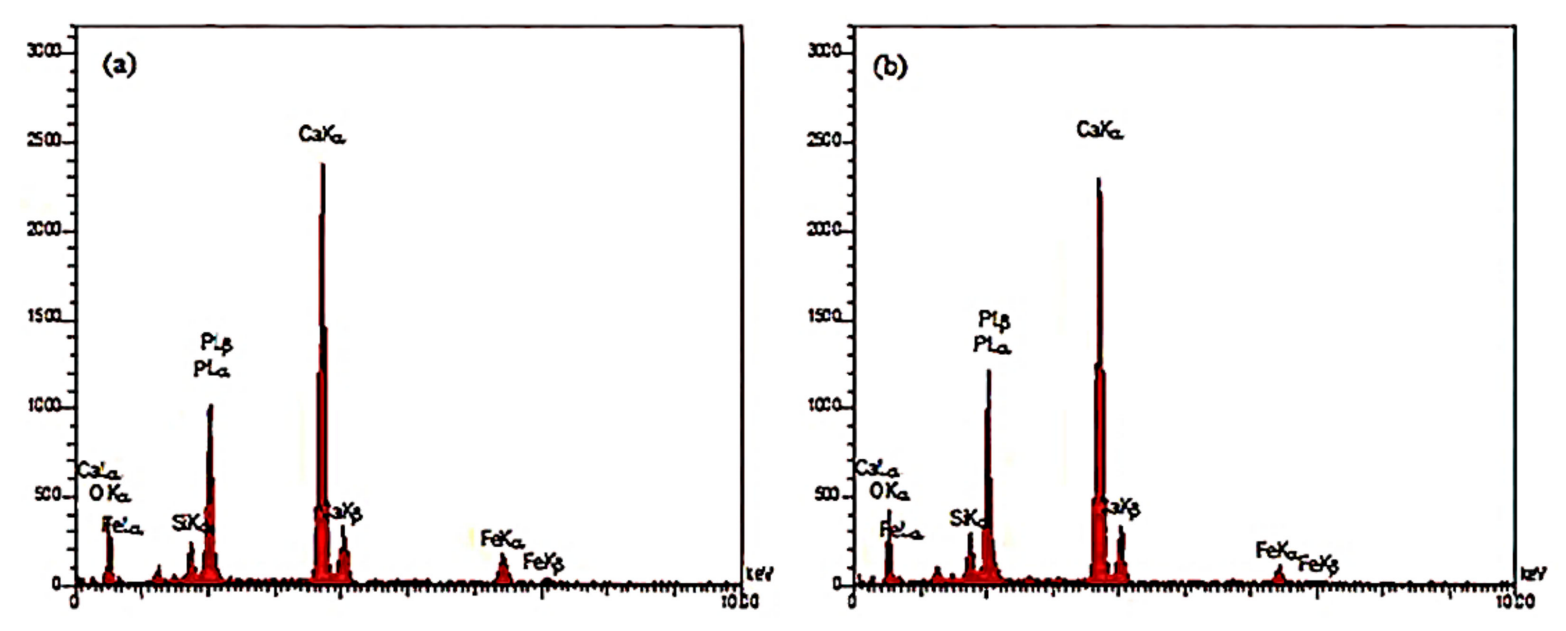
EDX spectra of: (**a**) NP and (**b**) 6-recycled NP.

EDX spectra in Figure 7 show that both NP and 6-recycled NP consist of constituent elements of Ca, P, Si, Fe and O [47,59]. As seen in Figure 7b, there is a marginal loss for Fe atoms during the reaction and recycling process. Two oxygen atoms in phosphate ion (PO_4_^3-^, P_2_O_5_) are coordinated, each one to a Fe^3+^ ion, creating a binuclear surface complex of Fe-O-P(O_2_)-O-Fe. The phenomenon of phosphate adsorption in Scheme 2 presents the creation route of this coordination structure on the surface of NP. Two surface oxygen groups of NP exist in intermediate **I** and **II** (shown in Scheme 3), by which the aminic hydrogen (NH) of diamine can form a doubly H-bonded composition to give hydroxyl (see structures 1 and 2, Scheme 2 and Scheme 3); subsequently, they are replaced with one oxygen of phosphate group (P_2_O_5_) of NP [60,61,62]. It is confirmed that gray-to-black-colored particles that appeared on the recycled NP is hematite (Fe_2_O_3_), which is released from NP during the reaction [63,64]. This can be the cause of the decreasing catalytic activity of recycled NP in the model reaction [65]. 

### 2.3. Synthesis of Quinoxaline

The reaction conditions were optimized for the condensation of 1,2-phenylenediamine **1a** (1 mmol) and benzil **2a** (1 mmol) in the model reaction. First, the reaction was carried out by using different protic and aprotic solvents (Table 3). The protic solvents showed better results than aprotic ones (Table 3, entries 1–4). Regarding the mechanism, the formation of a hydrogen bond between protic solvents (B-H) and the oxygen atom of the carbonyl groups of diketone compound increased the electrophilic character of carbonyl groups. Intramolecular nucleophilic attack at the carbon atom of carbonyl compound by the nitrogen atom of the amine group (via the intermediate **I**) was followed by intramolecular proton transfer in NP (see Scheme 3). Thus, the simultaneous activation of the carbonyl compound and protic solvents took place through the hydrogen bond formation with protic solvents. The time of the model reaction in H_2_O extended to a longer span than the other protic solvents, but their yields remained relatively unchanged because the reagents were not dissolved well in H_2_O (Table 3, entries 1 and 4) [58,66,67]. The best result was obtained from methanol, with the desired product **3a** collected with the yield of 98% (Table 3, entry **3**). This observation supported the involvement of hydrogen bond formation, where the solvation of the compounds increased with an increase in the polarity of the solvent. As the polarity of methanol was higher than ethanol, it acted as a better proton (H^+^) donor [68], being more effective in hydrogen bond formation with both solving reagent and activating carbonyl groups (intermediate **I** and **II**, shown in Scheme 3).

By varying the weight of NP (Table 4, entries 1–6), the model reaction progressed with a good yield of product 3a in the absence of a catalyst, so methanol could act as the co-catalyst (Table 4, entry 1). In terms of both reaction time and yield of product 3a, the best result was obtained by using 0.003 g of NP (Table 4, entry 4). The major constituent of NP is fluorapatite, so, experiments were carried out to verify the catalytic activity of fluorapatite. SFAP was prepared as explained in the experimental Section 3.2 [36] and used in the model reaction as the catalyst (Table 4, entries 7 and 8). SFAP was not as effective as NP under the same reaction conditions, as it required a higher quantity of catalyst and a longer reaction time (Table 4, entries 7 and 8). From the XRD analysis (Figure 2), the comparable catalytic activity of NP and SFAP was independent of lattice parameters and the average crystallite size of NP. The higher catalytic activity of NP was originated from fluorapatite in addition to the low content of some phases, such as coesite, SiO_2_ [48] and hematite, Fe_2_O_3_ [69]. So, we performed the model reaction in the presence of the prepared Fe_2_O_3_ and SiO_2_ in order to examine their catalytic effects (Table 4, entries 9 and 10). The obtained results confirmed the fingerprint of SiO_2_ and Fe_2_O_3_ in the catalytic activity. It is likely that the moderate incorporation of SiO_2_ and Fe_2_O_3_ resulted in the higher catalytic activity of NP than SFAP [65] (Table 4, entry 10). The oxygen atoms of CaO and P_2_O_5_ (fluorapatite) [70], SiO_2_ [71] and Fe_2_O_3_ [60] in NP acted as the bases to attract the hydrogen of primary amine through hydrogen bonding [14]. In addition to these, the active Lewis acidic sites of Fe_2_O_3_ [72] would play a key role in increasing the electrophilic properties of carbonyl groups. 

**Figure 8:**
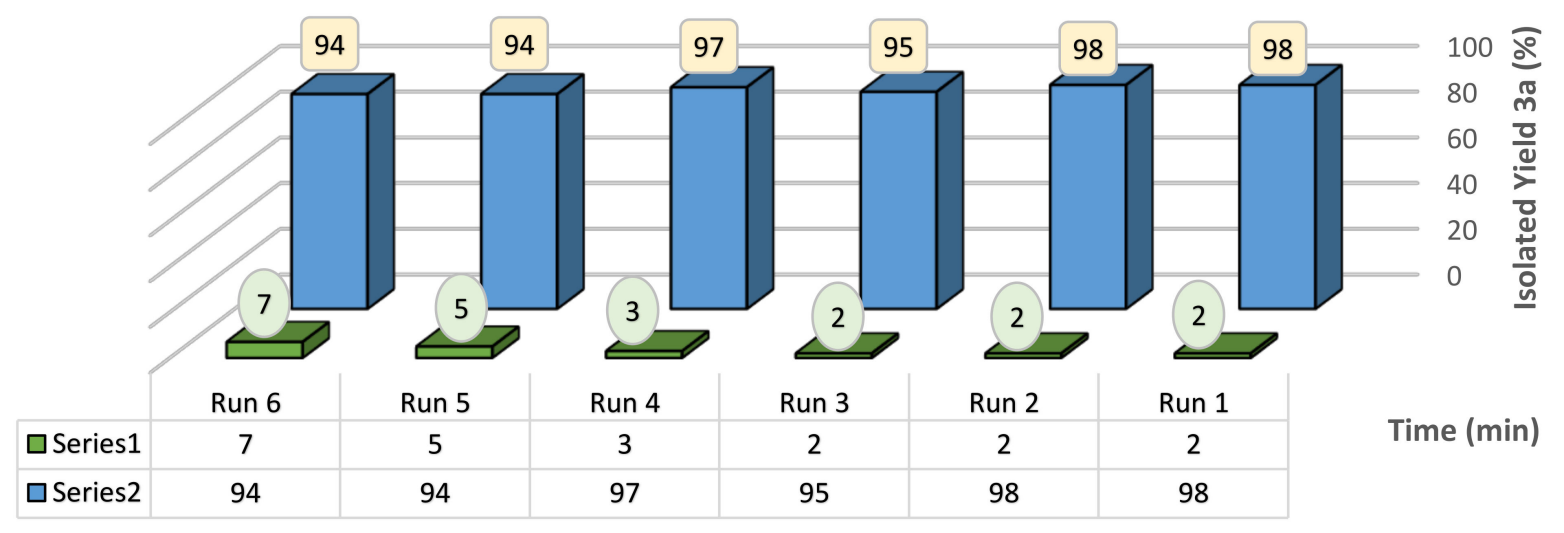
Recyclability of NP for the synthesis of quinoxaline **3a.** Reaction conditions: with 1,2-Penylen-diamine (1 mmol), benzil (1 mmol) and NP (0.003 g) in MeOH (5 mL) at room temperature.

After the first cycle with a 98% yield of quinoxaline **3a**, the catalyst was recovered by filtration, washing with acetone, drying and calcination at 900 °C in air for 30 min. Then, the reusability of NP was studied by examining the model reaction under optimized conditions (Figure 8). The results show that the catalyst could be recycled for at least six runs without significant loss of catalytic activity; as shown in Table 2, this could be due to decreasing the surface area and pore volume of recycled NP, which may facilitate the strong adsorption of a small amount of either quinoxaline **3a** or other unknown by-products. As mentioned earlier in Figure 7, decreasing the amount of Fe in the recycled NP could cause low yields of **3a** at the longer reaction times, in which Fe^3+^ acted as Lewis acid and catalyzed the reaction.

To assess the generality and scope of the proposed method, the optimized reaction conditions were applied to various 1,2-diamines and 1,2-dicarbonyl compounds (Table 5). The reactions proceeded efficiently at room temperature. The corresponding quinoxalines were obtained with high to excellent yields at short reaction times without any side reactions. 

Here, the activity and efficiency of catalysts for the synthesis of quinoxaline are compared with those previously reported (Table 6). Solid phase synthesis of quinoxaline using solid materials, such as K10 [73], alumina [74], silica [71] and graphite [75], needs longer reaction times or harsh reaction conditions (Table 6, entries 1–4). With a polymer as support, in addition to the multi-step preparation process (Table 6, entry 15), no data were found regarding the reusability of the catalyst (Table 6, entries 5–7). Other catalysts suffer from cost inefficiency and/or longer reaction times (Table 6, entry 8–13). NH_4_Cl results in high yield but it requires a larger quantity of catalyst for the reaction (Table 6, entry 14). Using MIL-101-Cr-NH-RSO_3_H and nanostructured pyrophosphate Na_2_PdP_2_O_7_ suffers from its difficult, multi-step and costly routes as well as extra material preparation (Table 6, entries 15 and 16). NP is a low-cost, readily available and natural solid catalyst which can be stored at ambient temperature without losing its catalytic potential after recycling by simple filtration (Table 6, entry 17), while SFAP needs more reaction time and catalyst (Table 6, entry 18).

After clarifying the role of NP, a mechanism is proposed in Scheme 3 for the facile formation of quinoxalines. The surface of NP presents multi-catalytic active sites (oxygen atoms of P_2_O_5_ or PO_4_ group and CaO) [85,86,87] where the aminic hydrogen (NH) of diamine (**1**) with the oxygens on the surface of NP and also protic solvent (B^_^H) can form doubly H-bonded motifs with the oxygen atoms of carbonyl group of diketone (Intermediate **I**). Enhancing the nitrogen electrophilicity of the aminic group of 1,2-diamine followed by the nucleophilic attack of amine on the carbonyl carbon (Intermediate **I**) [75,88], along with the protonation and subsequent dehydration (Intermediate **II**), ended with the production of quinoxaline **3** and regenerating NP. Thus, NP accelerated the cyclocondensation process beside the HB-assisted activation [89]. 

## 3. Materials and Methods

All chemicals were purchased from Merck, Germany, and Sigma Aldrich, USA. The structural data of all synthesized products were compared with the authentic data from the literature. The chemical composition of NP was determined using a PANalytical XRF spectrometer Venus 200 (Malvern PANalytical Ltd, Malvern, UK). The surface imaging was obtained by using an SEM, EM-3200 (KYKY Technology Development Ltd. Zhongguancun, Beijing, China). XRD were performed at room temperature on a Philips 1710 diffractometric goniometer (Philips Technology Inc., Amsterdam, The Netherlands), mounted with a Bragg–Brentano configuration (θ, 2θ) and using Ni-filtered Cu–K_α_ radiation (λ = 1.54 Å) along with PANalytical XPert HighScore Plus software (Malvern PANalytical Ltd., Malvern, UK). Surface areas were determined at 77 K using a Coulter SA 31000 instrument with an automated gas volumetric method by employing nitrogen as the adsorbate through the BET and BJH methods (Beckman Coulter Inc., Brea, CA, USA). The EDX pattern was attained by using a *stdLess* Rontec analyzer (TScan company, Waltham, MA, USA). The pH measurements were performed using a microprocessor pH meter (Metrohm, 827 pH Lab) (Metrohm AG, Herisau, Switzerland). Melting points were recorded by utilizing a Buchi B540 melting point apparatus. Fourier-transform infrared (FT-IR) spectra were recorded on a Bruker Perkin-Elmer spectrometer with KBr pellets for solids (Bruker company, Billerica, MA, USA). The ^1^H and ^13^C NMR spectra were recorded on a 400 MHz machine, Bruker Advance DRX spectrometer in CDCl_3_, using tetramethylsilane as an internal reference. 

### 3.1. Preparation of NP

NP was collected from a known ore extract site, the Bafgh region, in Yazd province, Iran. This kind of NP is used by various local industries to prepare phosphate fertilizer and phosphoric acid [7]. The extracted NP was pre-treated by refluxing in water, calcinating at 900 °C in air for 2 h, washing with water and finally re-calcinating at 900 °C in air for half an hour prior to usage as the catalyst [12].

### 3.2. Synthesis of SFAP

SFAP was prepared according to the method introduced by Sebti et al. [36]. A solution of di-ammonium hydrogen phosphate ((NH_4_)_2_HPO_4,_ 7.92 g), ammonium fluoride (NH_4_F, 1 g), and double distilled water (250 mL) was prepared for further usage. The pH of the solution was adjusted to pH > 12 by using ammonium hydroxide (NH_4_OH); subsequently, calcium nitrate solution (Ca(NO_3_)_2_·4H_2_O, 23.6 g) in double distilled water (150 mL) was slowly added to the mixture. The resultant suspension was refluxed for 4 h. The reaction mixture was cooled down to room temperature, and the solid particles were collected, washed with water and dried overnight at 80 **°**C. Finally, the dried sample was calcined at 800 °C in air for 1 h to obtain SFAP.

### 3.3. Measuring the Point of Zero Charge (pH_pzc_)

To show the basicity of the surface of NP, pH_pzc_ was measured. pHs of a series of 50 mL 0.01 M NaCl solutions were adjusted to values between 2 and 12 by adding HCl (0.1 M) or NaOH (0.1 M) solutions in a closed Erlenmeyer flask. The pH values of these solutions were recorded as initial pH (pH_I_). Then, 0.2 g of NP was added to them to reach the final pH (pH_F_) measured after 48 h. The values of pH_F_ vs. pH_I_ and pH_I_ vs. pH_I_ were plotted to obtain pH_pzc_ at their intersections [49].

### 3.4. Synthesis of Quinoxalines

In a typical reaction, a mixture of substituted cyclic 1,2-diamine **1** (1 mmol) and diketone **2** (1 mmol) was dissolved in methanol (5 mL). To this solution, NP (0.003 g) or SFAP (0.01 g) was added and stirred vigorously for 1–45 min for NP and 60–120 min for SFAP at room temperature. After completing the reaction, the mixture was filtered and washed with methanol (5 mL) in order to separate the catalyst. After the filtration, the methanolic filtrate was evaporated under vacuum to afford a crude product. Spectral data of obtained quinoxaline are presented in the Supplementary Information (SI).

## 4. Conclusions

The new use of natural phosphate was found as an efficient, inexpensive, recyclable and heterogeneous basic catalyst. This kind of NP could be stored at ambient temperature without majorly losing its catalytic potential after recycling by simple restoration filtering. NP catalyzed the condensation of 1,2-diamines with 1,2-diketones compounds in methanol at room temperature as an efficient way for the synthesis of quinoxaline derivatives with excellent yields in very short reaction time. The catalytic activity of the main component of natural phosphate was individually investigated. NP was characterized to be rich in fluorapatite as the major phase and minor phases of coesite (SiO_2_) and hematite (Fe_2_O_3_). So, the synthetic fluorapatite (SFAP), SiO_2_ and Fe_2_O_3_ were prepared, and their catalytic activities were compared with natural phosphate in the reaction. NP catalysed the quinoxaline reaction with the high yield and much shorter reaction time (0.003 g, 98% yield in 2 min (for **3a** product)) when compared to SFAP (0.01 g, 95% yield in 60 min), SiO_2_ (0.01 g, 98% yield in 120 min) and Fe_2_O_3_ (0.01 g, 98% yield in 150 min). NP had a low cost, a simple procedure, ready availability and good reusability for at least six runs without a major loss for the synthesis of quinoxalines at room temperature.

## Figures and Tables

**Table 1:** Average crystallite size of the samples.

Entry	Sample	Average Crystallite Size D (nm)
1	NP	155.76
2	SFAP	75.98

**Table 2:** Surface area, pore volume and average pore size of NP and 6-recycled NP.

Catalyst	Surface Area (m^2^/g)	Pore Volume (cm^3^/g)	Average Pore Size (nm)
NP	2.081 (BET) 1.948 (BJH)	0.007	3.675
6-recycled NP	0.761 (BET) 0.426 (BJH)	0.001	5.784

**Table 3:** Effect of various solvents on the synthesis of **3a** by using NP as the catalyst.

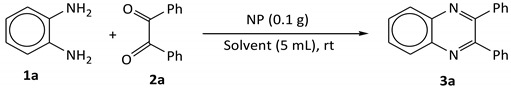			
Entry	Solvent	Time (min)	Isolated Yield 3a (%)
1	H_2_O	120	98
2	EtOH	30	95
3	MeOH	10	98
4	EtOH:H_2_O (1:1)	120	96
5	EtOAc	120	70
6	CH_3_CN	90	93
7	Et_2_O	30	91
8	1,4-dioxane	20	93
9	DMSO	30	91

**Table 4:** Effect of various amounts of catalyst on the synthesis of **3a**.

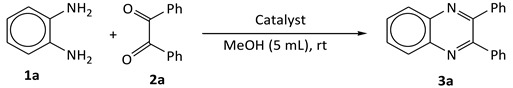				
Entry	Catalyst	Quantity of Catalyst (g)	Time (min)	Isolated Yield 3a (%)
1	-	-	120	70
2	NP	0.001	3	87
3	NP	0.002	2	90
4	NP	0.003	2	98
5	NP	0.004	2	98
6	NP	0.005	2	98
7	SFAP	0.005	90	95
8	SFAP	0.01	60	95
9	Fe_2_O_3_	0.01	150	98
10	SiO_2_	0.01	120	98

**Table 5:** Synthesis of quinoxalines 3 by using NP and SFAP as catalysts.

Entry	Diketone	Product 3	Time/Isolated Yield 3 (min/%)	
			NP	SFAP
1	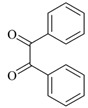	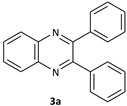	2/98	60/95
2	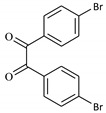	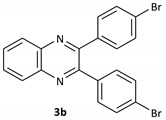	1/99	60/97
3	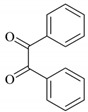	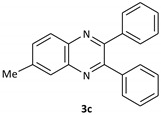	2/99	55/95
4	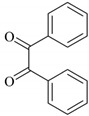	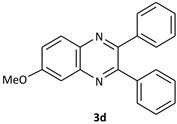	1/99	60/97
5	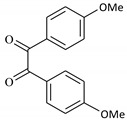	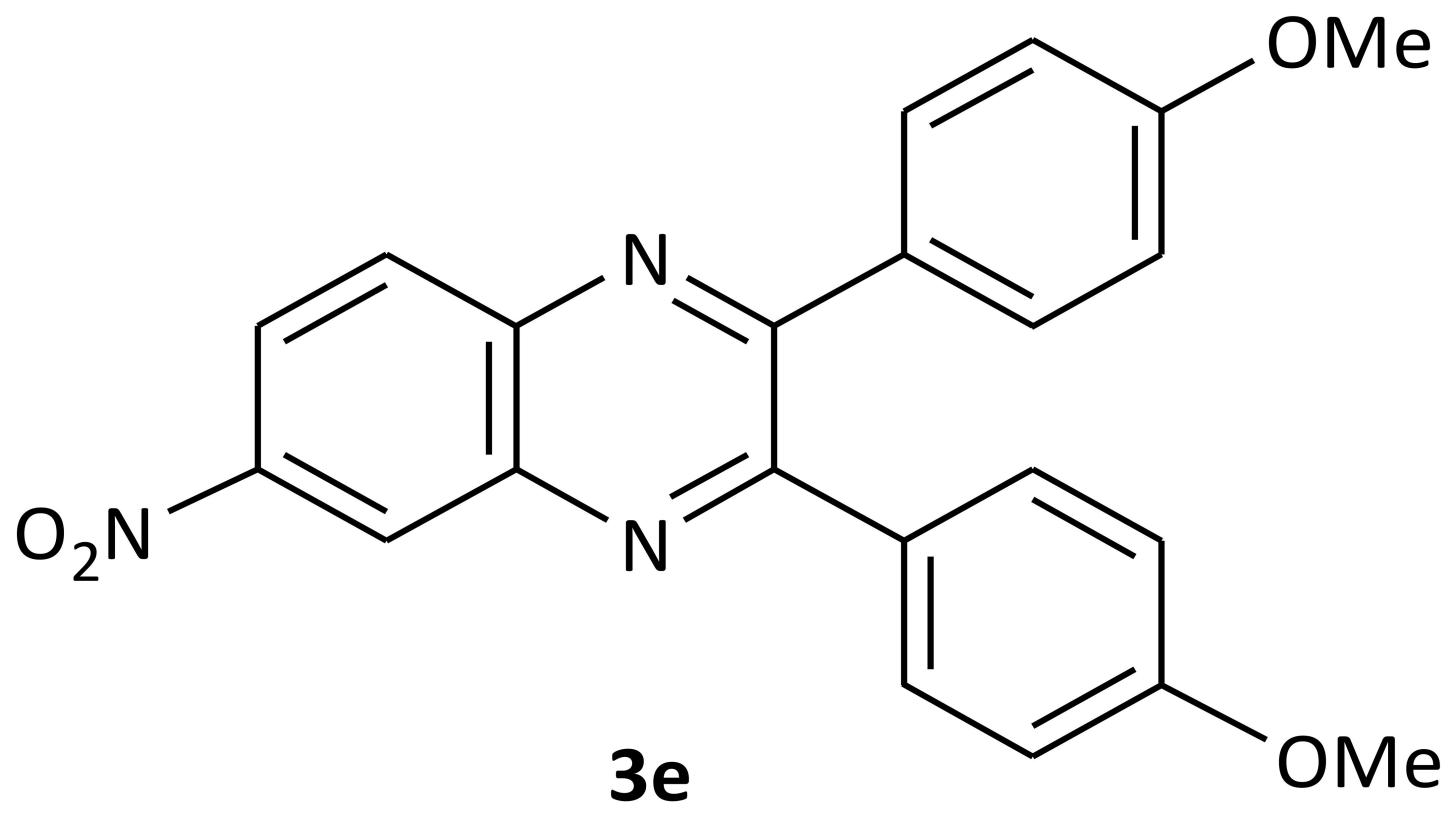	30/95	90/92
6	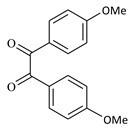	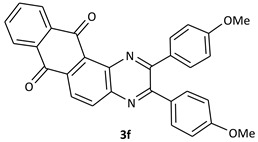	40/96	100/88
7	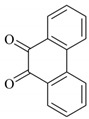	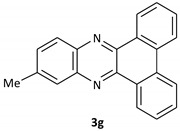	15/93	70/90
8	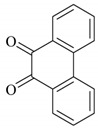	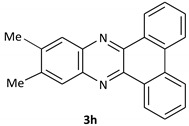	10/95	70/92
9	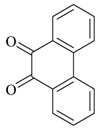	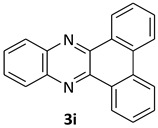	12/97	80/95
10	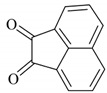	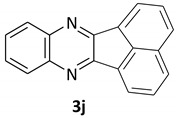	12/95	70/88
11	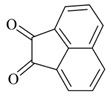	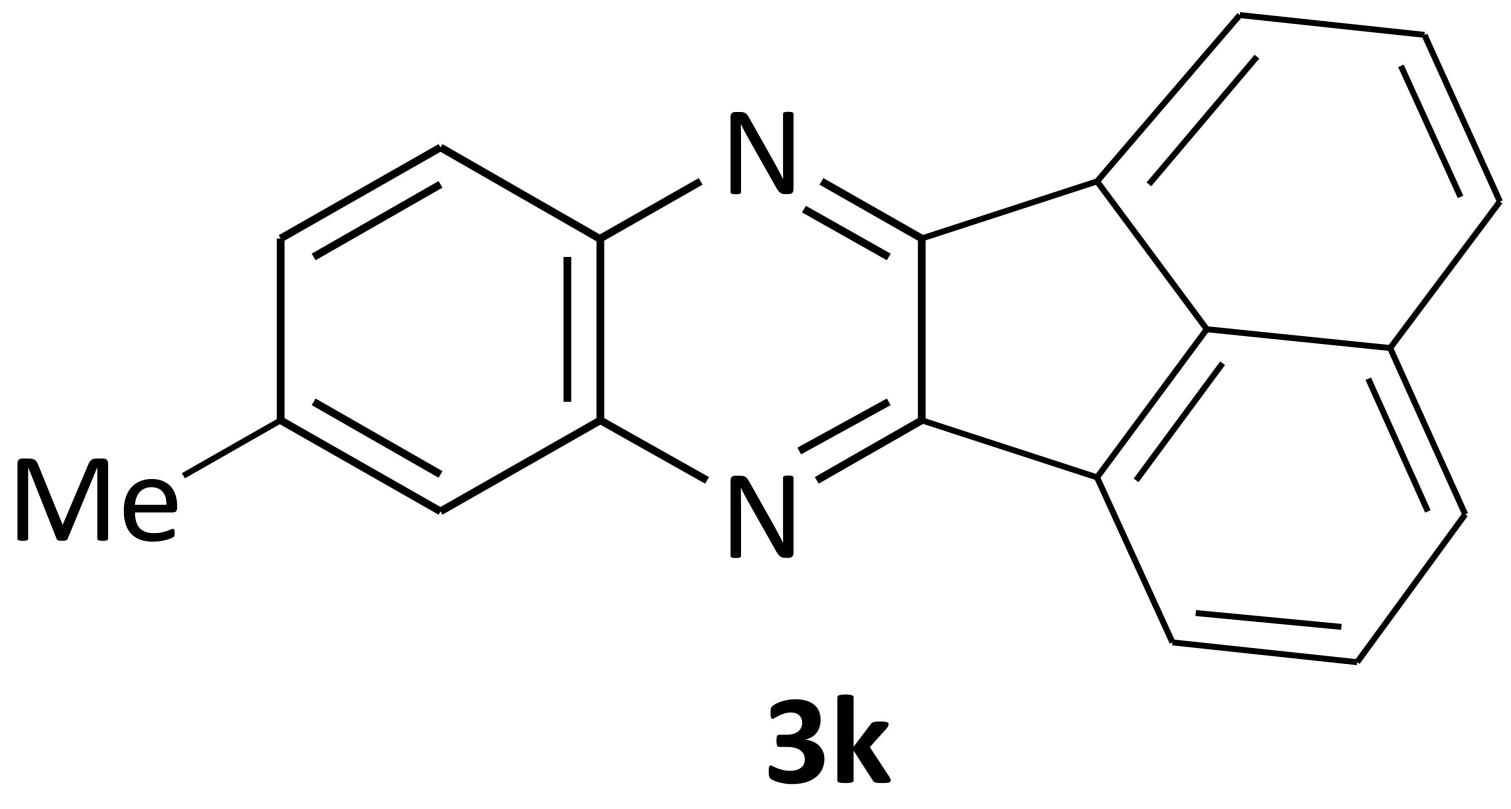	10/97	70/95
12	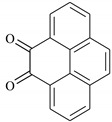	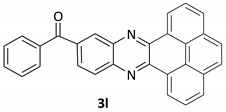	45/90	120/87
13	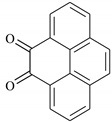	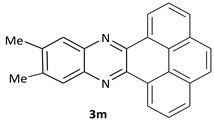	10/93	80/92
14	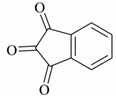	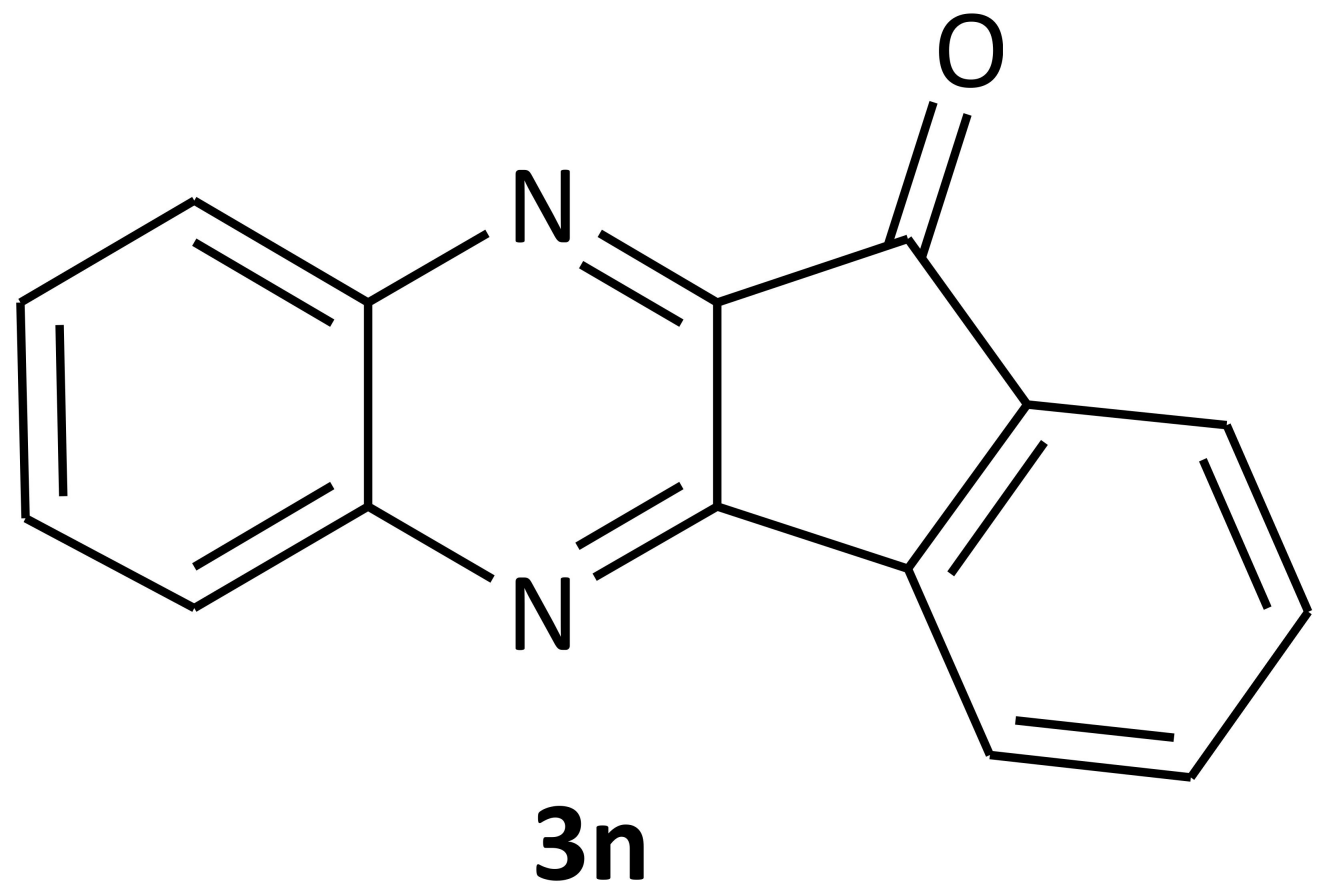	5/94	70/91
15	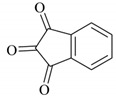	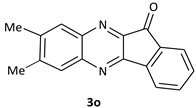	20/96	110/92
16	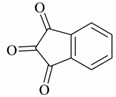	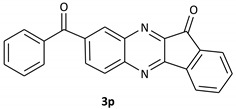	30/92	120/89

Reaction condition: 1,2-diamine (1 mmol), 1,2-diketone (1 mmol), methanol (5 mL), NP (0.003 g) or SFAP (0.01 g) at room temperature.

**Table 6:** Synthesis of quinoxaline using different catalysts.

Entry	Catalyst	Temperature/Solvent/Time (^°^C/-/min)	Yield 3a (%)	Reference
1	Mont K-10 (10 mol %)	25/H_2_O/150	100	[73]
2	Alumin ^a^ (0.2 g)	80/Solvent-free/2	96	[74]
3	Silica gel (1.0 g)	Grind,100/Solvent-free/45	94	[71]
4	Graphite (2 mmol)	rt/EtOH/60	92	[75]
5	Zr-CAP-SGa	rt/EtOH/90	92	[30]
6	SBA-Pr-SO_3_H ^b^ (0.02 g)	rt/CH_2_Cl_2_/10	95	[76]
7	PVPP.OTf ^c^ (30 mg)	rt/H_2_O/60	90	[77]
8	Nano ZrO_2_ (0.004 g)	60/EtOH/2	95	[78]
9	MnFe_2_O_4_ NP ^d^ (10 mol%)	rt/EtOH/150	91	[79]
10	Mn/Al_2_O_3_	50/EtOH:H2O/45	95	[80]
11	SbCl_3_/SiO_2_ (2.5 mol %)	rt/MeOH/60	98	[81]
12	ZrCl_4_ (5 mol%)	rt/ MeOH/240	98	[82]
13	SnCl_2_/SiO_2_ (5 mol%)	rt/ MeOH/4	100	[83]
14	NH_4_Cl (200 mol%)	rt/ MeOH/5	100	[84]
15	MIL-101-Cr-NH-RSO_3_H ^e^ (3.9 mol% -SO_3_H)	45/MeOH/5	91	[28]
16	Nanostructured pyrophosphate Na_2_PdP_2_O_7_	rt/ EtOH/30	98	[21]
17	NP (0.003 g)	rt/MeOH/2	98	**This study**
18	SFAP (0.01 g)	rt/MeOH/60	95	**This study**

Reaction conditions: 1,2-Penylen-diamine (1 mmol), benzil (1 mmol) and catalyst. ^a^ Zr (IV) modified on silica gel. ^b^ Sulfonic Acid Functionalized Nanoporous Silica (SBA-Pr- SO_3_H). ^c^ Polyvinylpolypyrrolidone supported triflic acid (PVPP.OTf). ^d^ MnFe_2_O_4_ nanoparticles. ^e^ Alkyl sulfonate functionalized metal organic frameworks (MOFs).

## Supplementary Materials

The following are available online at https://www.mdpi.com/article/10.3390/ijms222413665/s1.
