# Supplementary material for: Natural vs. Synthetic Phosphate as Efficient Heterogeneous Compounds for Synthesis of Quinoxalines
Source: Int J Mol Sci. 2021 Dec 20;22(24):13665. doi: 10.3390/ijms222413665 (PMC8704691; doi:10.3390/ijms222413665)
Supplement: Supplementary file 1 [file ijms-22-13665-s001.zip › ijms-1452036-supplementary.pdf]

Supporting Information

Natural vs. Synthetic Phosphate as Efficient Heterogeneous Compounds for Synthesis of Quinoxalines

Abbas Amini, Azadeh Fallah, Ahmad Sedaghat, Ahmad Gholami, Chun Cheng and Anju R. Gupta

---

Contents

Characterizations of Catalysts 2
XRD analysis of NP and SFAP 2
Spectral data for compounds 2
2, 3-Diphenylquinoxaline (3a) 2
2, 3-Bis-(4-bromo-phenyl)-quinoxaline (3b) 2
6-Methyl- 2, 3-diphenyl-quinoxaline (3c) 3
6-Methoxy-2, 3- diphenylquinoxaline (3d) 3
2, 3-Bis-(4-methoxy-phenyl)-6-nitro-quinoxaline (3e) 3
2, 3-bis-(4-methoxy-phenyl)-1, 4-diaza-benzo [a] antracene-7, 12-dione (3f) 3
11-methyl Dibenzo [a,c] phenazine (3g) 4
11, 12 -dimethyl Dibenzo [a,c] phenazine (3h) 4
Dibenzo [a,c] phenazine (3i) 4
6-Methyl acenaphtho [1, 2-b] quinoxaline (3k) 5
Phanantro [4, 5-abc] phenazine-11-yl-phenyl methanone (3l) 5
11, 12-dimethyl phanantro [4, 5-abc] phenazine (3m) 5
Indeno [1, 2-b] quinoxalin-11-one (3n) 5
6-methyl-Indeno [1, 2-b] quinoxalin-11-one (3o) 6
8-Benzoyl-11H-indeno- [1, 2- b] quinoxalin-11-one (3p) 6
1H NMR spectral figures 7
^{1}H NMR spectrum of 2, 3-Diphenylquinoxaline (3a) 7
^{1}H NMR spectrum of 2, 3-Bis-(4-bromo-phenyl)-quinoxaline (3b) 8
^{1}H NMR spectrum of 6-Methyl-2, 3-diphenyl-quinoxaline (3c) 9
^{1}H NMR spectrum of 6-Methoxy-2,3-diphenyl-quinoxaline (3d) 10
^{1}H NMR spectrum of 2, 3-Bis-(4-methoxy-phenyl)-6-nitro-quinoxaline (3e) 11
^{1}H NMR spectrum of 2, 3-bis-(4-methoxy-phenyl)-1, 4-diaza-benzo [a] antracene-7, 12-dione (3f) 12
^{1}H NMR spectrum of 11-methyl Dibenzo [a,c] phenazine (3g) 13
^{1}H NMR spectrum of 11, 12 -dimethyl Dibenzo [a, c] phenazine (3h) 14
^{1}H NMR spectrum of Dibenzo[a,c]phenazine (3i): 15
^{1}H NMR spectrum of Acenaphtho [1, 2-b] quinoxaline (3j) 16
^{1}H NMR spectrum of 6-Methyl acenaphtho [1, 2-b] quinoxaline (3k) 17
^{1}H NMR spectrum of 2, 3-Bis-(4-methoxy-phenyl)-6-nitro-quinoxaline (3l) 18
^{1}H NMR spectrum of 2, 3-Bis-(4-methoxy-phenyl)-6-nitro-quinoxaline (3m) 19
^{1}H NMR spectrum of Indeno [1, 2-b] quinoxalin-11-one (3n) 20
^{1}H NMR spectrum of 2, 3-Bis-(4-methoxy-phenyl)-6-nitro-quinoxaline (3o) 21
^{1}H NMR spectrum of 2, 3-Bis-(4-methoxy-phenyl)-6-nitro-quinoxaline (3p) 22
^{13}C NMR spectral figures 23

^{13}C NMR spectrum of 2, 3-Diphenylquinoxaline (3a) 23
^{13}C NMR spectrum o of 2, 3-Bis-(4-bromo-phenyl)-quinoxaline (3b) 24
^{13}C NMR spectrum of 6-Methyl-2, 3-diphenyl-quinoxaline (3c) 25
^{13}C NMR spectrum of 6-Methoxy-2,3-diphenyl-quinoxaline (3d) 26
^{13}C NMR spectrum of 2, 3-Bis-(4-methoxy-phenyl)-6-nitro-quinoxaline (3e) 27
^{13}C NMR spectrum of 2, 3-bis-(4-methoxy-phenyl)-1, 4-diaza-benzo [a] antracene-7, 12-dione (3f) 28
^{13}C NMR spectrum of 11, 12 -dimethyl Dibenzo [a,c] phenazine (3g) 29
^{13}C NMR spectrum of 11, 12 -dimethyl Dibenzo [a, c] phenazine (3h) 30
^{13}C NMR spectrum of Dibenzo[a,c]phenazine (3i): 31
^{13}C NMR spectrum of Acenaphtho [1, 2-b] quinoxaline (3j) 32
^{13}C NMR spectrum of 6-Methyl acenaphtho [1, 2-b] quinoxaline (3k) 33
^{13}C NMR spectrum of 2, 3-Bis-(4-methoxy-phenyl)-6-nitro-quinoxaline (3l) 34
^{13}C NMR spectrum of 2, 3-Bis-(4-methoxy-phenyl)-6-nitro-quinoxaline (3m) 35
^{13}C NMR spectrum of Indeno [1, 2-b] quinoxalin-11-one (3n) 36
^{13}C NMR spectrum of 2, 3-Bis-(4-methoxy-phenyl)-6-nitro-quinoxaline (3o) 37
^{13}C NMR spectrum of 2, 3-Bis-(4-methoxy-phenyl)-6-nitro-quinoxaline (3p) 38
References 38

Characterizations of Catalysts

XRD analysis of NP and SFAP

The XRD patterns of NP and some other standards are shown in Fig. S1. The obtained results of NP reveal the existence of three phases of which the major phase is fluorapatite [1, 2] (Fig. S1b) and two other phases are Coesite, SiO_2 [3] (Fig. S1c) and Hematite, Fe_2O_3 [4, 5] (Fig. S1d).

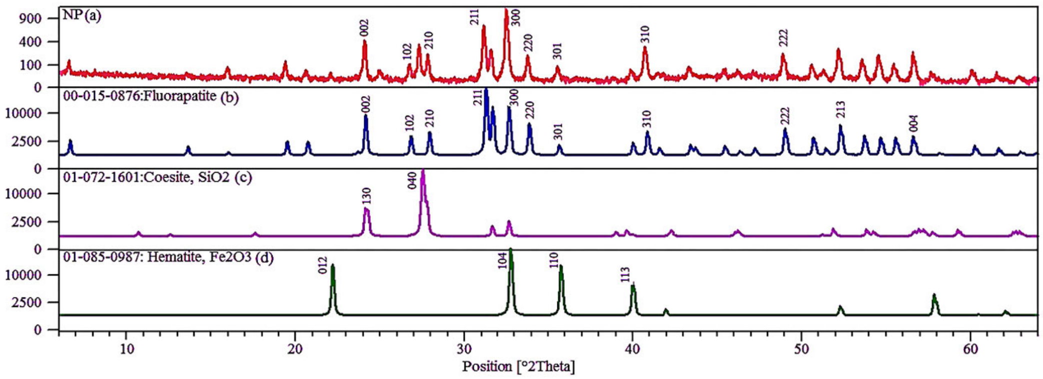

Figure S1. XRD patterns of NP and standards

Spectral data for compounds

2, 3-Diphenylquinoxaline (3a)

m. p. = 127 – 129 °C (reported 129 - 130) [6]; ^{1}H NMR (400 MHz, $CDCl_3$), δ: 7.34 – 7.41 (m, 6H), 7.52 – 7.57 (m, 4H), 7.78 – 7.83 (m, 2H), 8.19 – 8.23 (m, 2H); ^{13}C NMR (100 MHz, $CDCl_3$), δ: 128.29, 128.90, 129.22, 129.85, 129.98, 139.09, 141.24, 153.49. Elemental analysis, Found, %: C 85.03; H 5.02; N 9.86. $C_{20}H_{14}N_2$. Calculated, %: C 85.08; H 5.00; N 9.92.

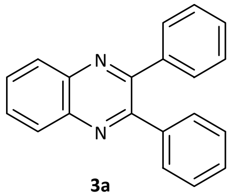

3a

2, 3-Bis-(4-bromo-phenyl)-quinoxaline (3b)

m. p. = 188 – 190 °C (reported 191 - 192) [7]. ^{1}H NMR (400 MHz, $CDCl_3$), δ: 7.42 (d, J = 8.4 Hz, 4H), 7.53 (d, J = 8.4 Hz, 4H), 7.80 – 7.84 (m, 2H), 8.16 – 8.20 (m, 2H). ^{13}C NMR (100 MHz, $CDCl_3$), δ: 123.72, 129.21, 130.45, 131.44, 131.71, 137.67, 141.25, 151.93. Elemental analysis, Found, %: C 54.46; H 2.71; N 6.31. $C_{20}H_{12}Br_2N_2$. Calculated, %: C 54.58; H 2.75; N 6.36.

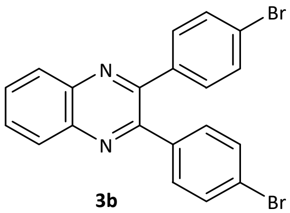

3b

6-Methyl- 2, 3-diphenyl-quinoxaline (3c)

m. p. = 112-115°C (reported 113-115) [7]; ^{1}H NMR (400 MHz, $CDCl_3$), δ: 2.64 (s, 3H), 7.32-7.38 (m, 6H), 7.51-7.54 (m, 4H), 7.63 (dd, J = 8.6, 1.6 Hz, 1H), 7.98 (s, 1H), 8.10 (d, J = 8.4 Hz, 1H); ^{13}C NMR (100 MHz, $CDCl_3$), δ: 21.94, 128.03, 128.24, 128.64, 128.70, 128.71, 129.83, 129.85, 132.32, 139.23, 139.72, 140.51, 141.30, 152.59, 153.34. Elemental analysis, Found, %: C 84.99; H 5.49; N 9.39 for $C_{21}H_{16}N_2$. Calculated, %: C 85.11; H 5.44; N 9.45.

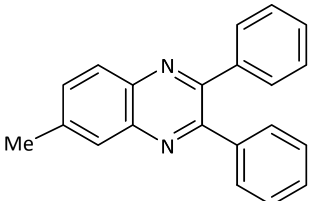

3c

6-Methoxy-2, 3- diphenylquinoxaline (3d)

m. p. = 154 - 155 °C (reported 156 - 157) [8]; ^{1}H NMR (400 MHz, $CDCl_3$), δ: 4.01 (s, 3H), 7.33 - 7.38 (m, 6H), 7.44 (dd, J = 9.2, 2.8 Hz, 1H), 7.50 – 7.54 (m, 5H), 8.08 (d, J = 9.2, 1H); ^{13}C NMR (100 MHz, $CDCl_3$), δ: 106.46, 123.34, 128.24, 128.27, 128.48, 128.71, 129.82, 130.18, 137.42, 139.20, 139.23, 142.74, 150.95, 153.34, 160.93. Elemental analysis, Found, %: C 80.78; H 5.11; N 8.92 for $C_{21}H_{16}N_2O$. Calculated, %: C 80.75; H 5.16; N 8.97.

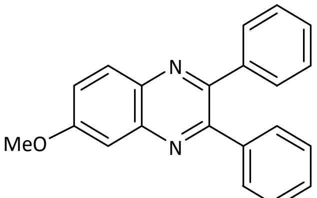

3d

2, 3-Bis-(4-methoxy-phenyl)-6-nitro-quinoxaline (3e)

m. p. = 188 – 189 °C (reported 188 - 190) [6]; ^{1}H NMR (400 MHz, $CDCl_3$), δ: 3.87 and 3.88 (s, 6H), 6.91 - 6.94 (m, 4H), 7.56 - 7.60 (m, 4H), 8.23 - 8.25 (d, J = 9.2 Hz, 1H), 8.48 - 8.51 (dd, J = 9.2, 2.6 Hz, 1H), 9.03 - 9.04 (d, J = 2.6 Hz, 1H); ^{13}C NMR (100 MHz, $CDCl_3$): δ: 55.38, 113.97, 122.88, 125.45, 130.43, 130.54, 130.54, 130.66, 131.34, 131.49, 139.75, 143.51, 147.56, 155.19, 155.79, 160.84,. Elemental analysis, Found, %: C 68.18; H 4.39; N 10.81. For $C_{22}H_{17}N_3O_4$. Calculated, %: C 68.21; H 4.42; N 10.85.

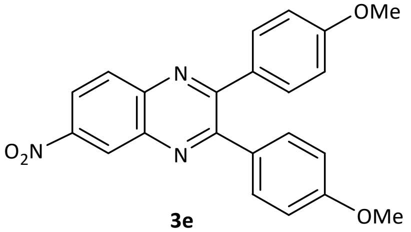

3e

2, 3-bis-(4-methoxy-phenyl)-1, 4-diaza-benzo [a] antracene-7, 12-dione (3f)

m. p. = 304 – 306 °C (reported 305 - 308) [6]; ^{1}H NMR (400 MHz, $CDCl_3$), δ: 3.87 (s, 6H), 6.96 (m, 4H), 7.70 (d, J = 8.0 Hz, 2H), 7.83 (d, J = 8.0 Hz, 2H), 7.96 (d, J = 8.4 Hz, 2H), 8.3 (dd, J = 16.8, 6.8 Hz, 1H), 8.51 (dd, J = 75.6, 8.4 Hz, 1H); ^{13}C NMR (100 MHz, $CDCl_3$), δ:. 55.39, 113.78, 114.04, 126.22, 127.32, 129.55, 130.59, 131.02, 131.34, 131.75, 131.92, 132.23, 132.39, 133.50, 134.48, 134.70, 134.85, 135.17, 138.36, 142.90, 153.72, 154.61, 161.01, 164.85, 183.52, 183.77, 193.51; Elemental analysis, Found, %: C 76.34; H 4.31; N 5.86 for $C_{30}H_{20}N_2O_4$. Calculated, %: C 76.26; H 4.27; N 5.93.

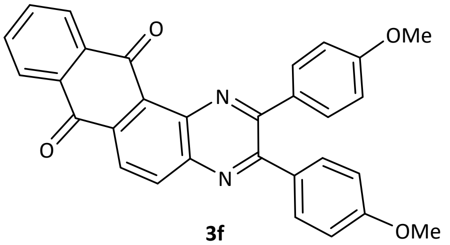

3f

11-methyl Dibenzo [a,c] phenazine (3g)

m. p. = 204 – 207 °C (reported 205 - 207) [7]. ^{1}H NMR ($CDCl_3$), δ: 2.7 (s, 3H)., 7.68 - 7.80 (m, 5H), 8.10 - 8.23 (m, 2H), 8.57 (d, J = 7.6 Hz, 2H), 9.39 (d, J = 4.8 HZ, 2H), ^{13}C NMR ($CDCl_3$), δ: 22.03, 122.65, 126.03, 126.16, 127.85, 127.87, 128.02, 128.93, 130.01, 130.14, 130.41, 131.81, 132.0, 132.41, 140.36, 140.79, 141.62, 142.27. Elemental analysis, Found, %: C 85.54; H 4.74; N 9.46 for $C_{30}H_{20}N_2O_4$. Calculated, %: C 85.69; H 4.79; N 9.52.

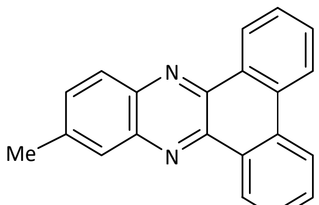

3g

11, 12 -dimethyl Dibenzo [a,c] phenazine (3h)

m. p. = 283 – 284 °C (reported 280 - 282) [9]; ^{1}HNMR (400 MHz, $CDCl_3$), δ: 2.58 (s, 6H, CH_3), 7.75 - 7.79 (m, 4H, Ar), 8.06 (s, 2H, Ar), 8.57 (d, J = 7.2 Hz, 2H, Ar), 9.38 (d, J = 7.2 Hz, 2H); ^{13}CNMR (100 MHz, $CDCl_3$), δ: 20.54, 122.85, 123.92, 125.97, 127.78, 128.22, 129.54, 129.85, 130.50, 130.56, 131.76, 135.97, 140.64, 141.63, Elemental analysis, Found, %: C 85.61; H 5.19; N 9.06 for $C_{22}H_{16}N_2$. Calculated, %: C 85.69; H 5.23; N 9.08.

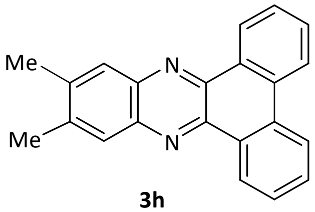

3h

.

Dibenzo [a,c] phenazine (3i)

m. p. = 216 – 221 °C; (Reported 224) [10]; ^{1}HNMR (400 MHz, $CDCl_3$), δ: 7.74 - 7.83 (m, 4H, Ar), 7.86 - 7.87 (m, 2H, Ar), 8.33 - 8.34 (m, 2H, Ar), 8.57 (d, J = 8.0 Hz, 2H), 9.41 (d, J = 7.6 HZ, 2H); ^{13}CNMR (100 MHz, $CDCl_3$), δ: 122.92, 126.26, 127.94, 129.45, 129.76, 130.31, 132.05, 142.19, 142.45. Elemental analysis, Found, %: C 85.53; H 4.29; N 9.93 for $C_{20}H_{12}N_2$. Calculated, %: C 85.69; H 4.31; N 9.99.

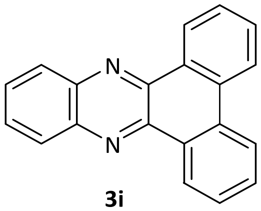

3i

Acenaphtho [1, 2-b] quinoxaline (3j)

m. p. = 241 – 243 °C (reported 242 - 244) [11]; ^{1}H NMR ($CDCl_3$), δ: 7.76 – 7.79 (m, 2H, Ar), 7.84 (dd, *J* = 7.2, 6.8Hz, 2H, Ar), 8.10 (d, *J* = 8.0 Hz, 2H, Ar), 8.21 – 8.24 (m, 2H, Ar), 8.42 (d, *J* = 7.2 Hz, 2H, Ar); ^{13}C NMR ($CDCl_3$), δ:

121.88, 128.67, 129.24, 129.49, 129.59, 130.01, 131.8, 136.51, 141.26, 154.08. Elemental analysis, Found, %: C 85.09; H 3.91; N 10.93 for $C_{18}H_{10}N_2$. Calculated, %: C 85.02; H 3.96; N 11.02.

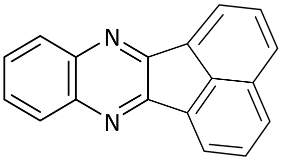

3j

6-Methyl acenaphtho [1, 2-b] quinoxaline (3k)

m. p. = 234 – 236 °C (reported 236 - 237) [11]; ^{1}H NMR (400 MHz, $CDCl_3$), δ: 2.6 (s, 3H), 7.52 (d, J = 8.4 Hz, 1H, Ar), 7.77 (t, J = 7.6 Hz, 2H, Ar), 7.91 (s, 1H, Ar), 8.01-8.05 (m, 3H, Ar), 8.33 (t, J = 6.0 Hz, 2H, Ar); ^{13}C NMR (100 MHz, $CDCl_3$), δ: 30.81, 121.57, 121.73, 128.60, 128.63, 128.98, 129.24, 129.39, 129.93, 131.31, 131.85, 136.18, 139.05, 139.72, 141.16, 153.25, 153.96. Elemental analysis, Found, %: C, 84.99; H, 4.49; N, 10.41 for $C_{19}H_{12}N_2$. Calculated, %: C, 85.05; H, 4.51; N, 10.44.

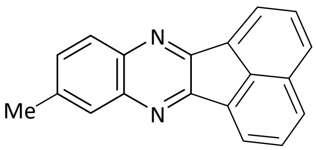

3k

Phanantro [4, 5-abc] phenazine-11-yl-phenyl methanone (3l)

m. p. = 188 – 190 °C (reported 191 - 192); ^{1}H NMR (400 MHz, $CDCl_3$), δ: 7.60 (m, 2H), 7.71 (m, 1H, Ar), 7.77 (m, 2H, Ar), 7.84 (m, 2H, Ar), 7.99 (m, 2H, Ar), 8.35 (dd, J = 7.6, 2.0 Hz, 1H, Ar), 8.43 (dd, J = 9.2, 0.4 Hz, 1H, Ar), 8.5 (m, 2H, Ar), 8.69 (d, 1H, Ar), 9.33 (dd, J = 8, 1.6 Hz, 1H, Ar), 9.42 (dd, J = 8, 1.6 Hz, 1H, Ar); ^{13}C NMR (100 MHz, $CDCl_3$), δ: 123.01, 126.35, 126.68, 128.12, 128.58, 129.40, 129.90, 129.93, 130.23, 130.75, 130.99, 132.16, 132.47, 132.84, 132.95, 137.37, 137.9, 141.03, 143.45, 143.74, 143.84, 153.25, 196.01. Elemental analysis, Found, %: C, 85.25; H, 3.92; N, 6.82 for $C_{29}H_{16}N_2O$ Calculated, % C, 85.28; H, 3.95; N, 6.86.

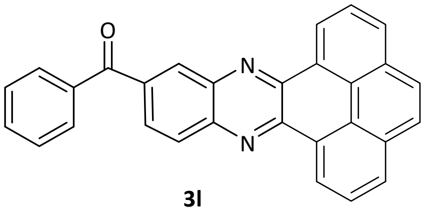

3l

11, 12-dimethyl phanantro [4, 5-abc] phenazine (3m)

m. p. = 297 – 300 °C (reported 236 - 237) [11]; ^{1}H NMR (400 MHz, $CDCl_3$), δ: 2.06 (s, 6H), 7.78 (m, 4H, Ar), 8.09 (s, 2H, Ar), 8.59 (d, J = 7.6 Hz, 2H, Ar), 9.40 (d, J = 8.0 Hz, 2H, Ar); ^{13}C NMR (100 MHz, $CDCl_3$), δ: 20.56, 122.87, 125.98, 127.80, 128.24, 129.87, 130.57, 131.78, 140.67, 141.35, 141.67. Elemental analysis, Found, %: C, 86.99; H, 4.81; N, 8.37 for $C_{24}H_{16}N_2$ Calculated, % C, 86.72; H, 4.85; N, 8.43.

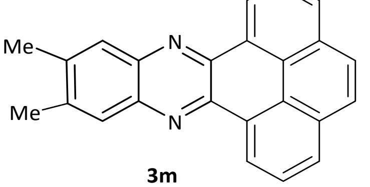

3m

Indeno [1, 2-b] quinoxalin-11-one (3n)

m. p. = 218 – 221 °C (reported 221 - 225) [11]; ^{1}H NMR (400 MHz, $CDCl_3$), δ: 7.60 - 7.64 (td, J = 6.8, 1.2 Hz, 1H, Ar), 7.75 - 7.81 (m, 2H, Ar), 7.82 - 7.86 (m, 1H, Ar), 7.95(d, J = 12 Hz, 1H, Ar), 8.12 (dd, J = 3.8, 0.4 Hz, 1H, Ar), 8.14

(dd, J = 8, 0.8 Hz, 1H, Ar), 8.26 (dd, J = 6.2, 0.8 Hz, 1H, Ar), ^{13}C NMR (100 MHz, $CDCl_3$), δ: 122.56, 124.82, 129.67, 130.32, 131.63, 132.49, 132.55, 136.70, 136.85, 141.57, 142.67, 143.14, 149.30, 156.64, 189.96 ppm. Elemental analysis, Found, %: C, 78.22; H, 3.44; N, 12.15 for $C_{15}H_8N_2O$: Calculated, % C, 77.58; H, 3.47; N, 12.06.

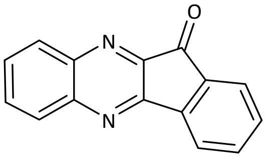

3n

6-methyl-Indeno [1, 2-b] quinoxalin-11-one (3o)

m. p. = 248 – 249 °C (reported 248) [12]; ^{1}HNMR (400 MHz, $CDCl_3$), δ: 2.51 (s, 3H), 2.53 (s, 3H), 7.58 (td, J = 7.6 , 1.2 Hz, 1H, Ar), 7.75 (td, J = 7.6 , 1.2 Hz, 1H, Ar), 7.88 (s, 1H, Ar), 7.91 (d, J = 7.6 Hz, 1H, Ar), 7.97 (s, 1H, Ar), 8.07 (d, J = 7.6 Hz, 1H, Ar); ^{13}CNMR (100 MHz, $CDCl_3$), δ: 20.32, 20.64, 122.16, 124.62, 129.03, 130.76, 132.07, 136.42, 136.60, 140.95, 141.51, 141.77, 141.97, 143.63, 148.31, 156.16, 190.36. Elemental analysis, Found, %: C, 78.38; H, 4.61; N, 10.69 for $C_{17}H_{12}N_2O$: Calculated, % C, 78.44; H, 4.65; N, 10.76.

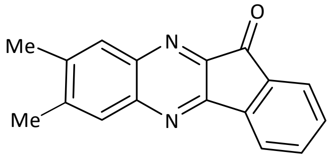

3o

8-Benzoyl-11H-indeno- [1, 2- b] quinoxalin-11-one (3p)

mp = 235-237°C (reported 238-240) [13]; ^{1}HNMR (400 MHz, $CDCl_3$), δ: 7.57 (t, J = 8.4 Hz, 2H, Ar), 7.65-7.71 (m, 2H, Ar), 7.85 (t, J = 8.4 Hz, 2H, Ar), 7.91 (dd, J = 8.0, 1,2 Hz, 2H, Ar), 7.98 (dd, J = 7.6, 1,2 Hz, 1H, Ar), 8.19 (dt, J = 7.6, 1,2 Hz, 1H, Ar), 8.26 (d, J = 8.8 Hz, 1H, Ar), 8.36 (dd, J = 8.4, 1.6 Hz, 1H, Ar), 8.62 (d, J = 1.6 Hz, 1H, Ar); ^{13}CNMR (100 MHz, $CDCl_3$), δ: 123.01, 124.99, 128.6, 130.13, 130.16, 132.45, 133.10, 133.21, 134.37, 136.73, 136.97, 137.10, 138.43, 141.18, 141.72, 145.11, 158.10, 189.28, 195.04. Elemental analysis, Found, %: C, 78.38; H, 3.64; N, 8.59 for $C_{22}H_{12}N_2O_2$: Calculated, % C, 78.56; H, 3.60, N, 8.33.

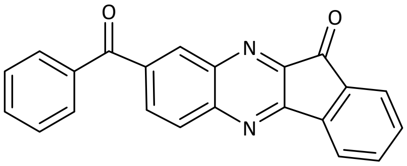

3p

1H NMR spectral figures

^{1}H NMR spectrum of 2, 3-Diphenylquinoxaline (3a)

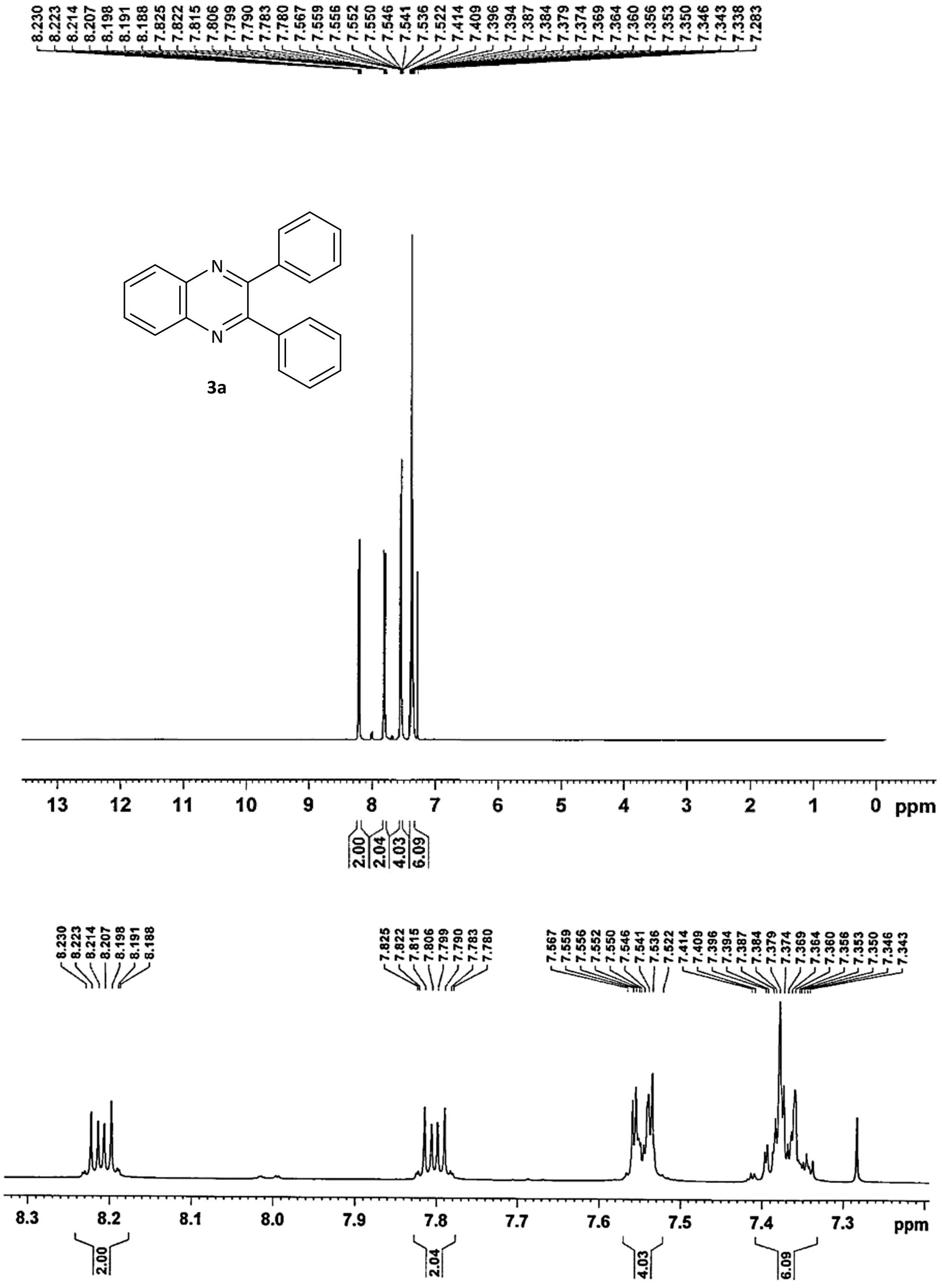

^{1}H NMR spectrum of 2, 3-Bis-(4-bromo-phenyl)-quinoxaline (3b)

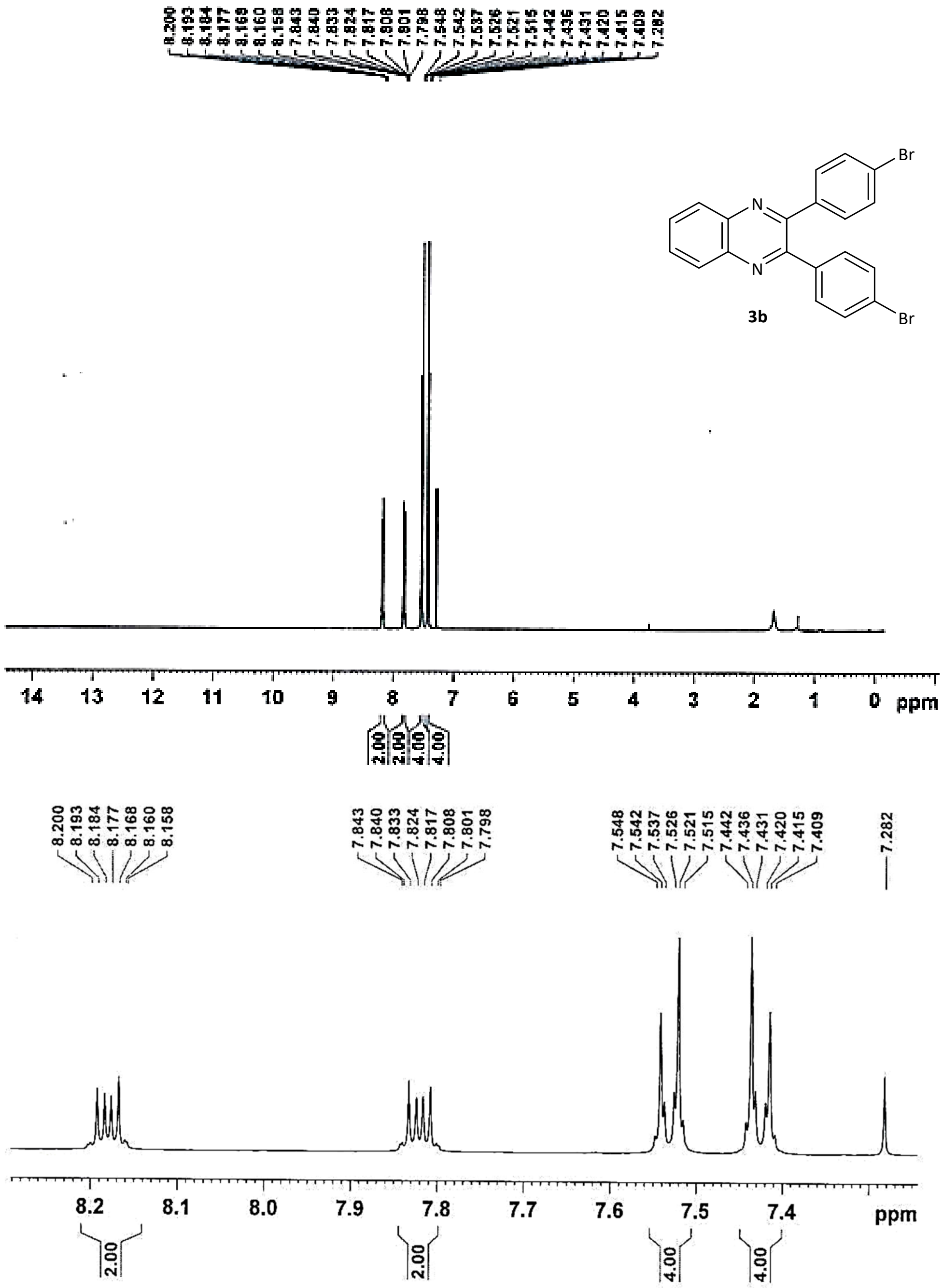

^{1}H NMR spectrum of 6-Methyl-2, 3-diphenyl-quinoxaline (3c)

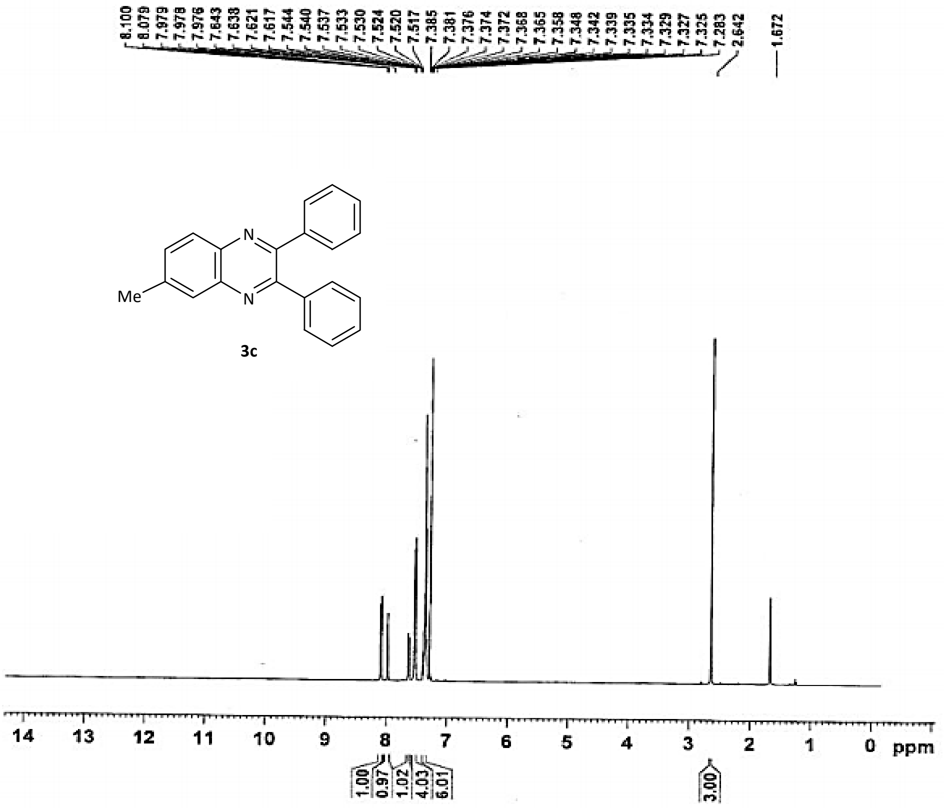

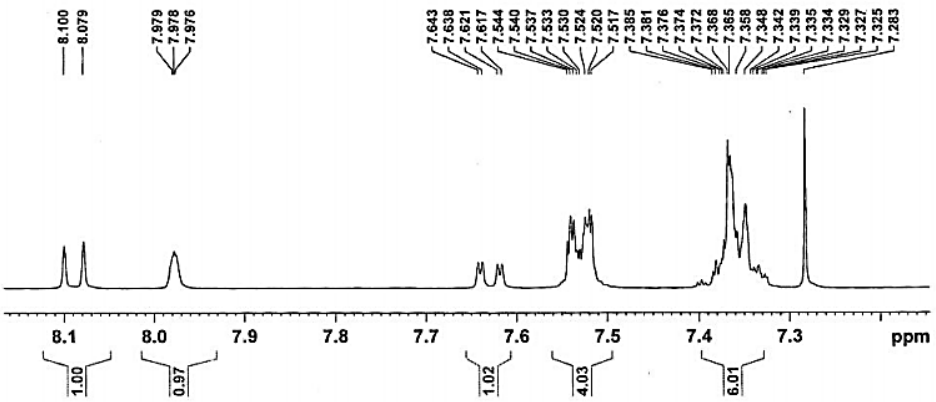

^{1}H NMR spectrum of 6-Methoxy-2,3-diphenyl-quinoxaline (3d)

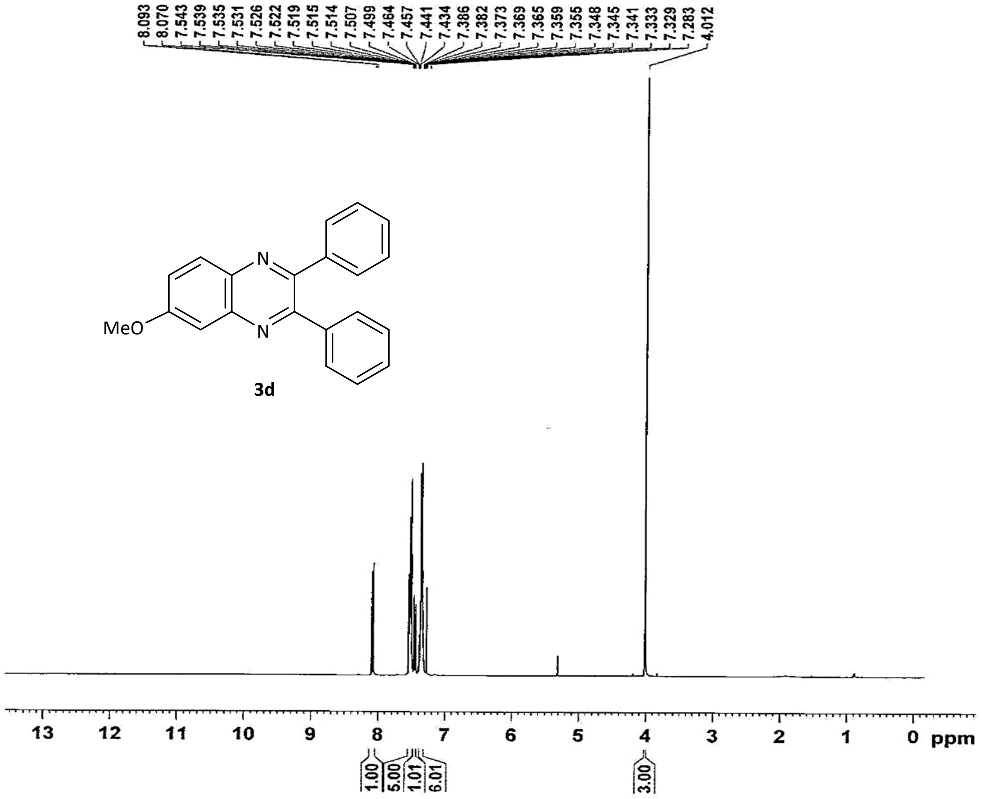

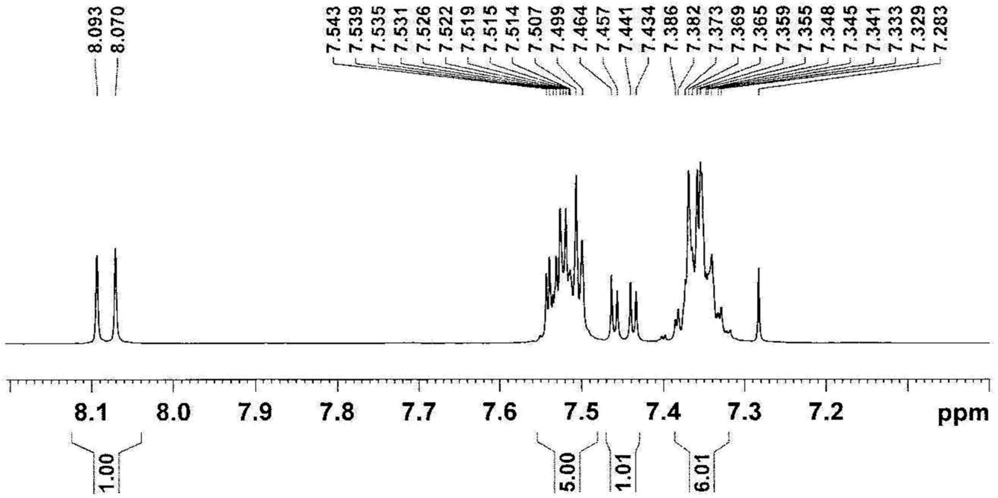

^{1}H NMR spectrum of 2, 3-Bis-(4-methoxy-phenyl)-6-nitro-quinoxaline (3e)

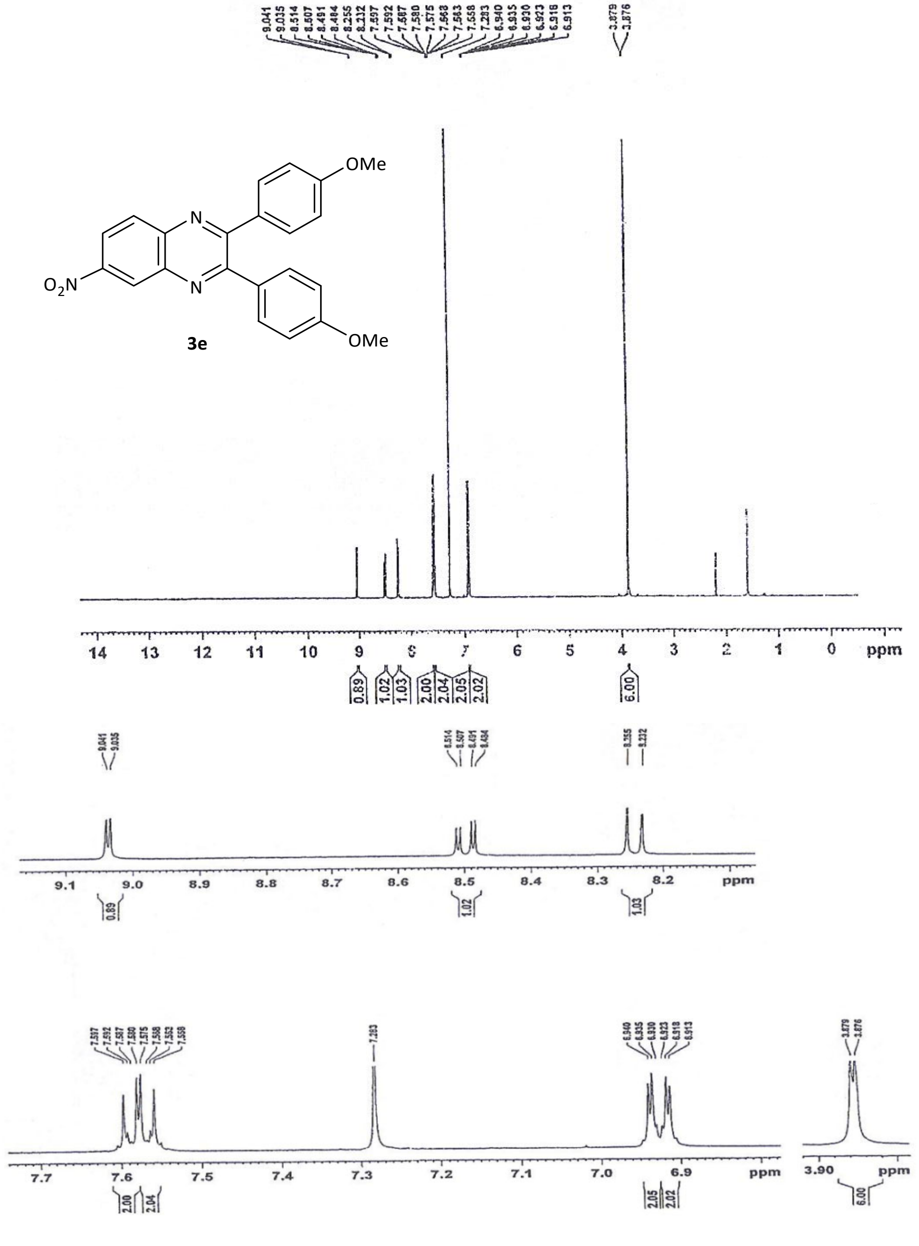

^{1}H NMR spectrum of 2, 3-bis-(4-methoxy-phenyl)-1, 4-diaza-benzo [a] antracene-7, 12-dione (3f)

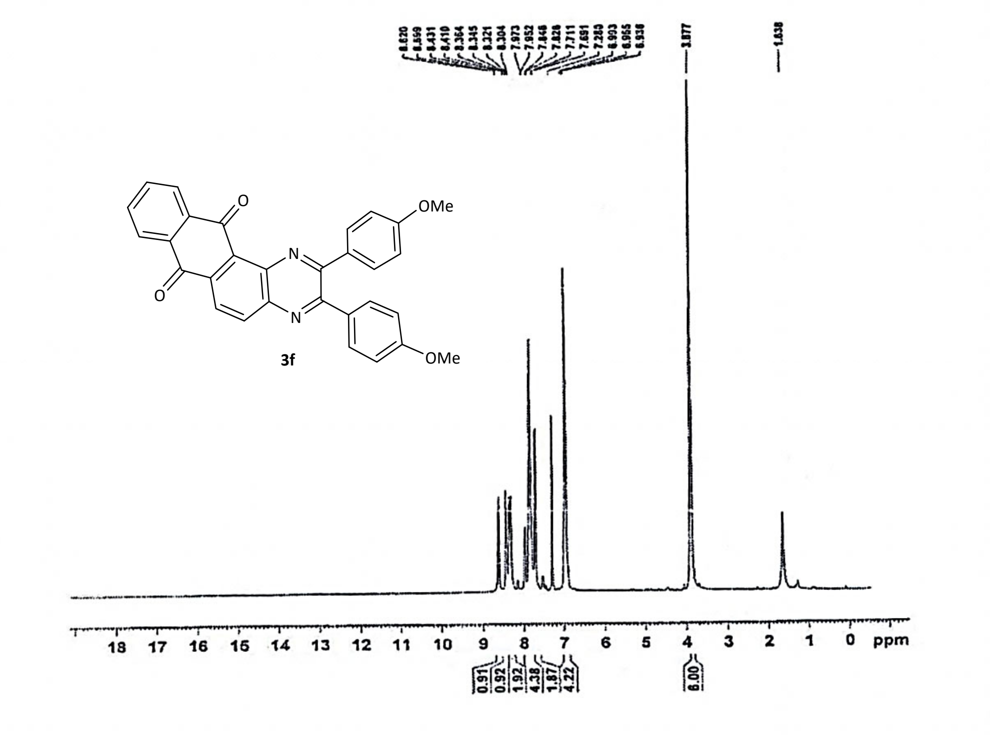

^{1}H NMR spectrum of 11-methyl Dibenzo [a,c] phenazine (3g)

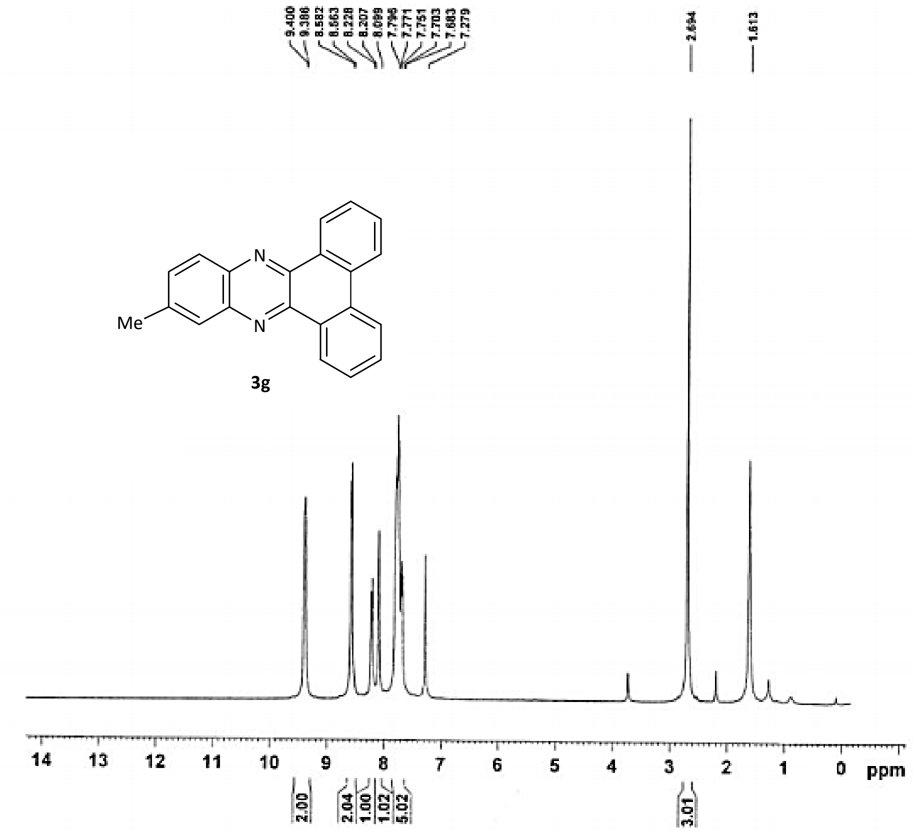

^{1}H NMR spectrum of 11, 12 -dimethyl Dibenzo [a, c] phenazine (3h)

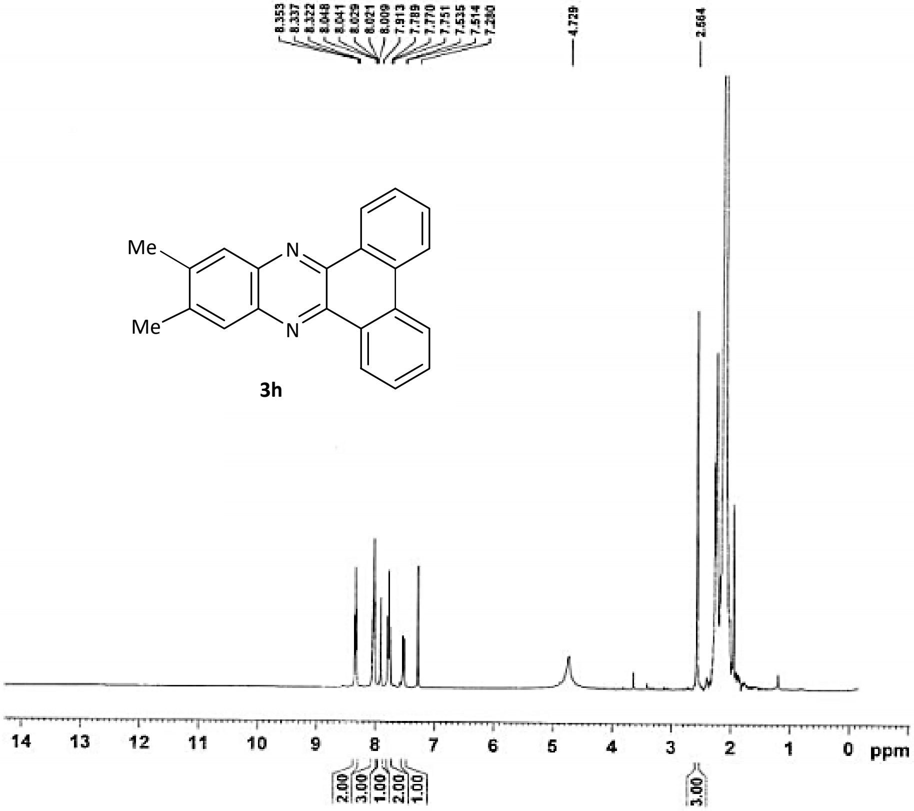

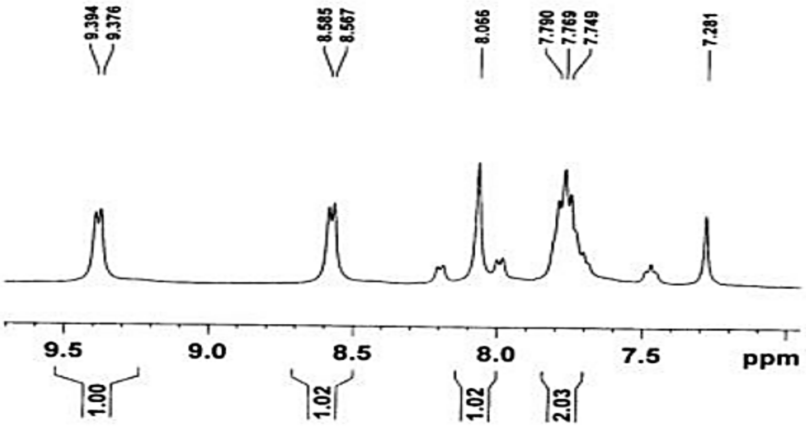

^{1}H NMR spectrum of Dibenzo[a,c]phenazine (3i):

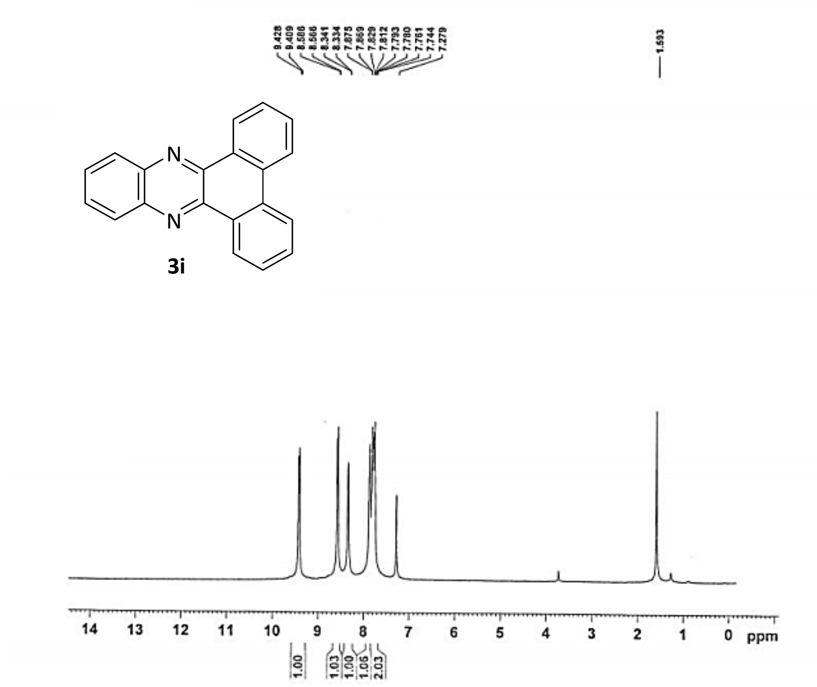

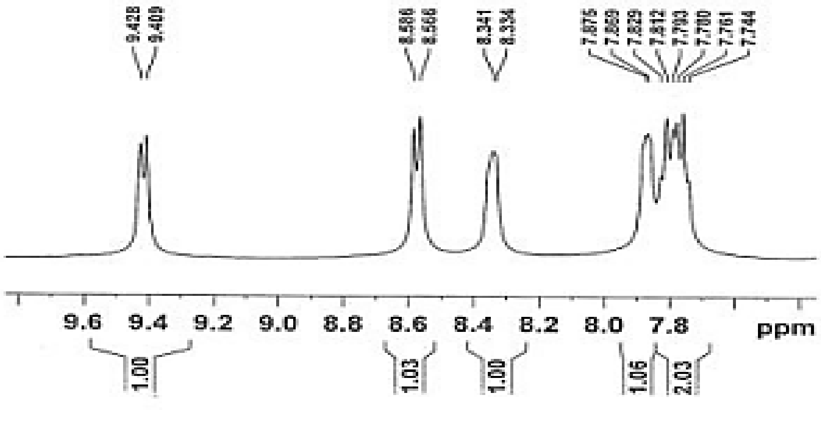

^{1}H NMR spectrum of Acenaphtho [1, 2-b] quinoxaline (3j)

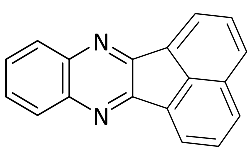

3j

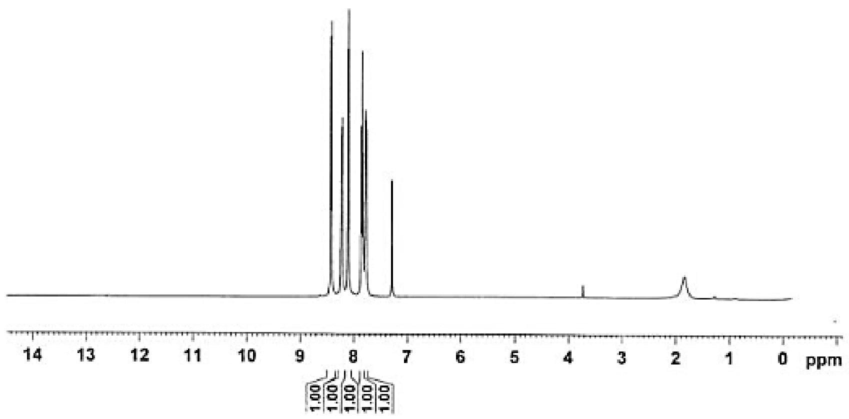

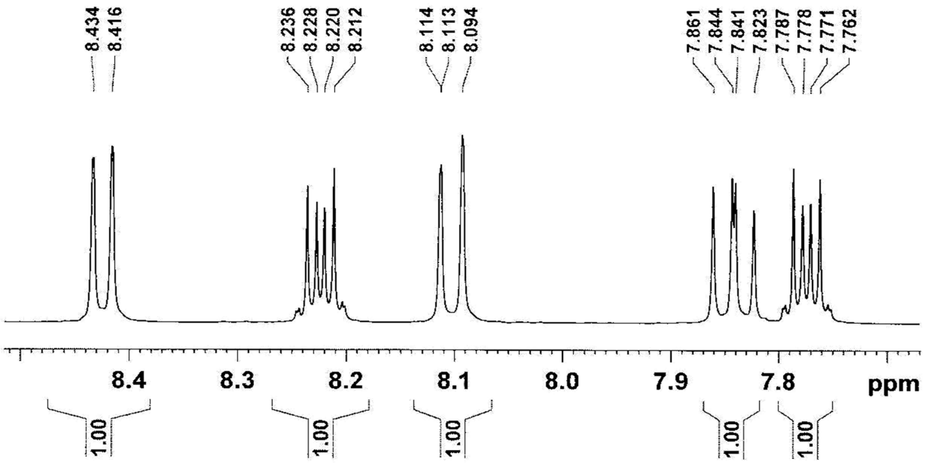

^{1}H NMR spectrum of 6-Methyl acenaphtho [1, 2-b] quinoxaline (3k)

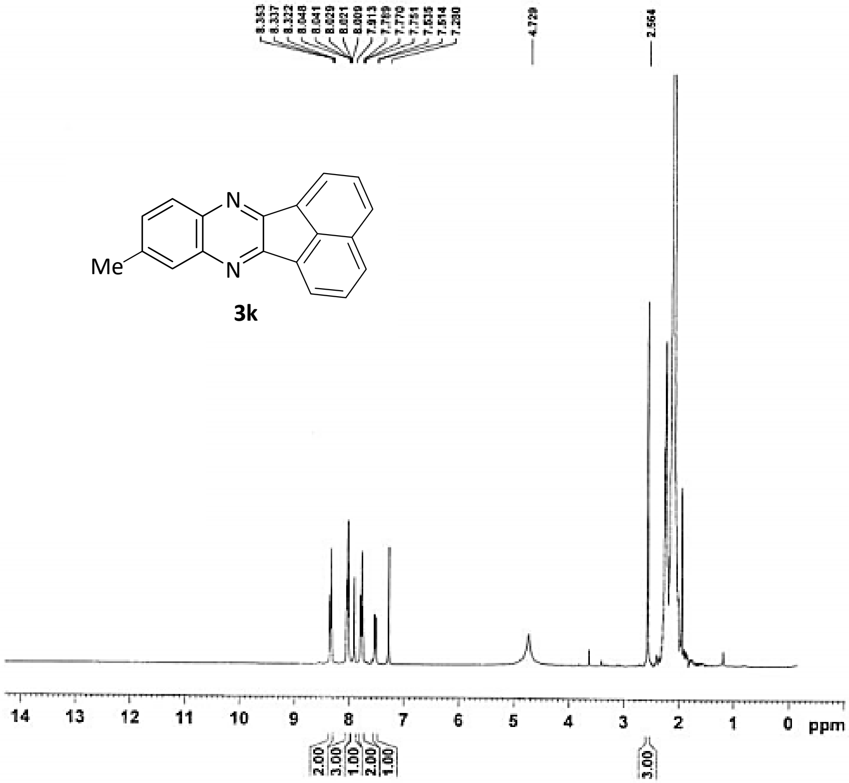

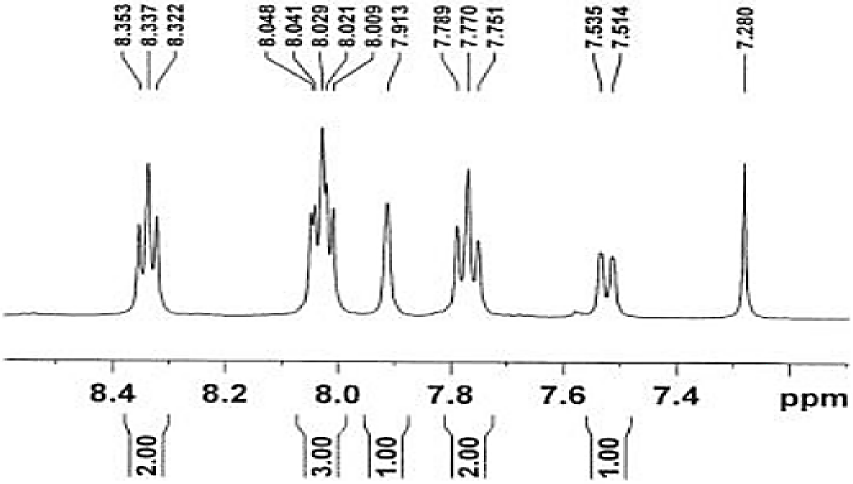

¹H NMR spectrum of 2, 3-Bis-(4-methoxy-phenyl)-6-nitro-quinoxaline (3l)

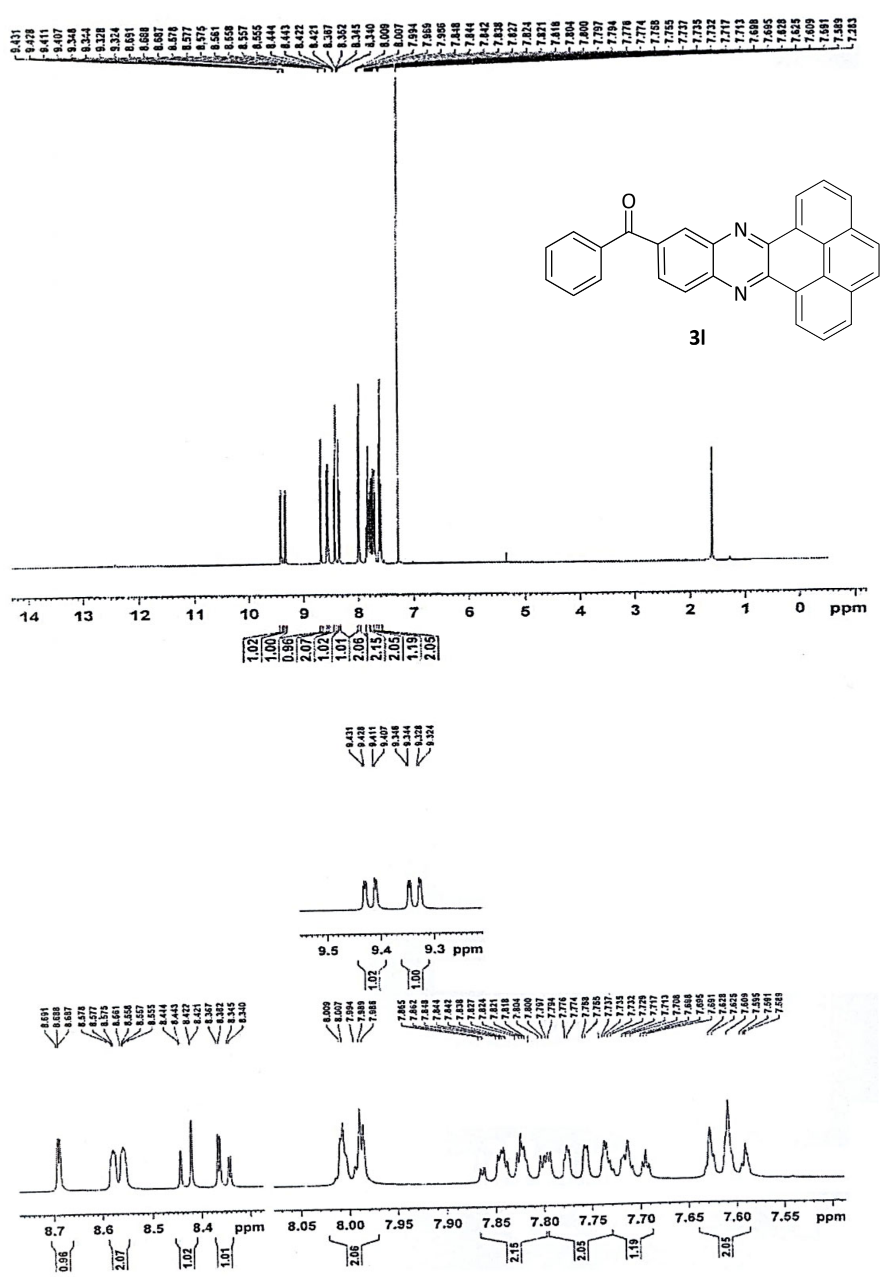

^{1}H NMR spectrum of 2, 3-Bis-(4-methoxy-phenyl)-6-nitro-quinoxaline (3m)

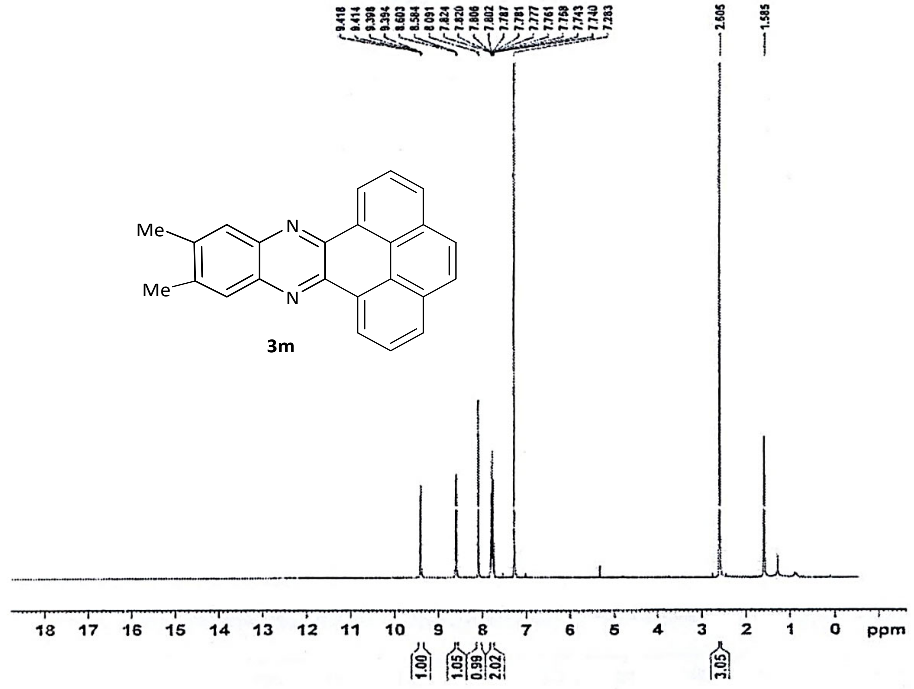

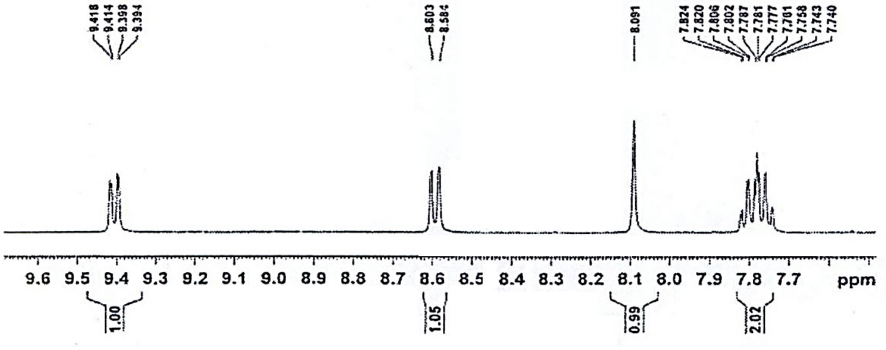

^{1}H NMR spectrum of Indeno [1, 2-b] quinoxalin-11-one (3n)

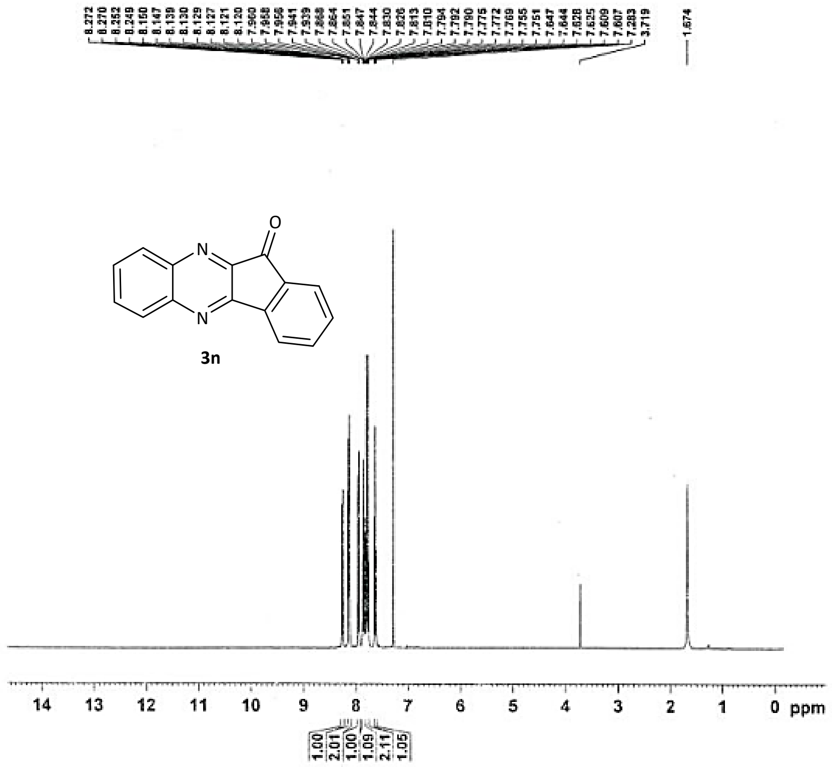

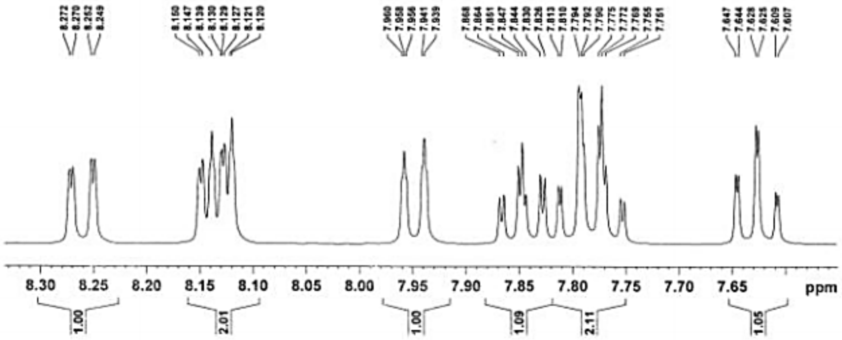

^{1}H NMR spectrum of 2, 3-Bis-(4-methoxy-phenyl)-6-nitro-quinoxaline (3o)

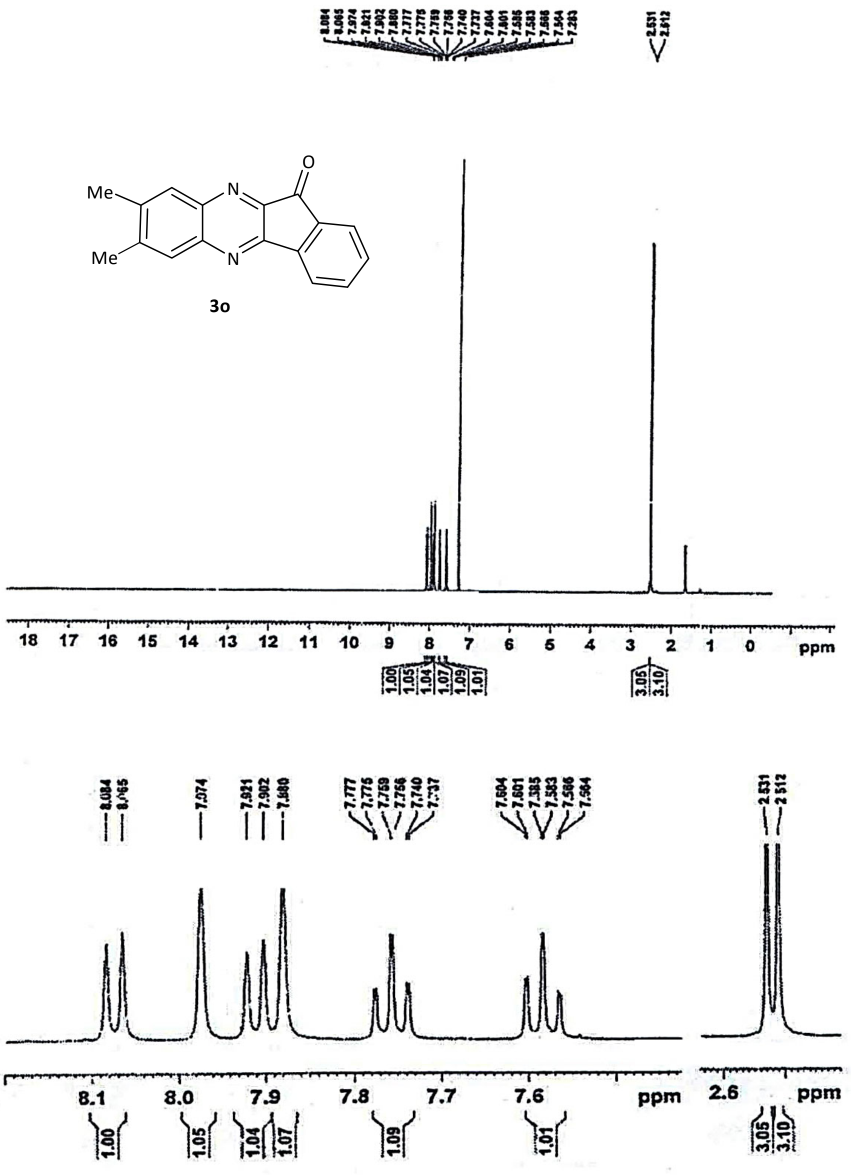

^{1}H NMR spectrum of 2, 3-Bis-(4-methoxy-phenyl)-6-nitro-quinoxaline (3p)

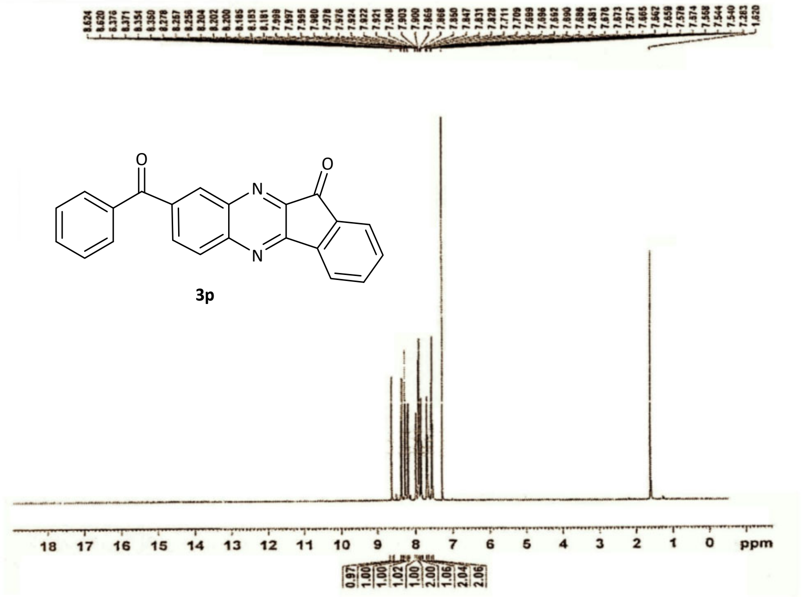

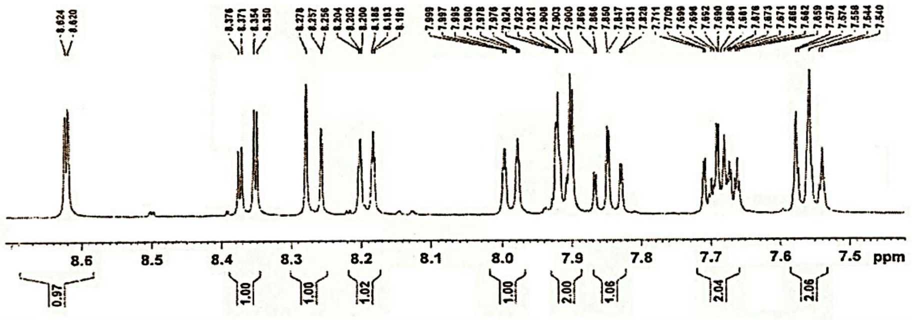

^{13}C NMR spectral figures

^{13}C NMR spectrum of 2, 3-Diphenylquinoxaline (3a)

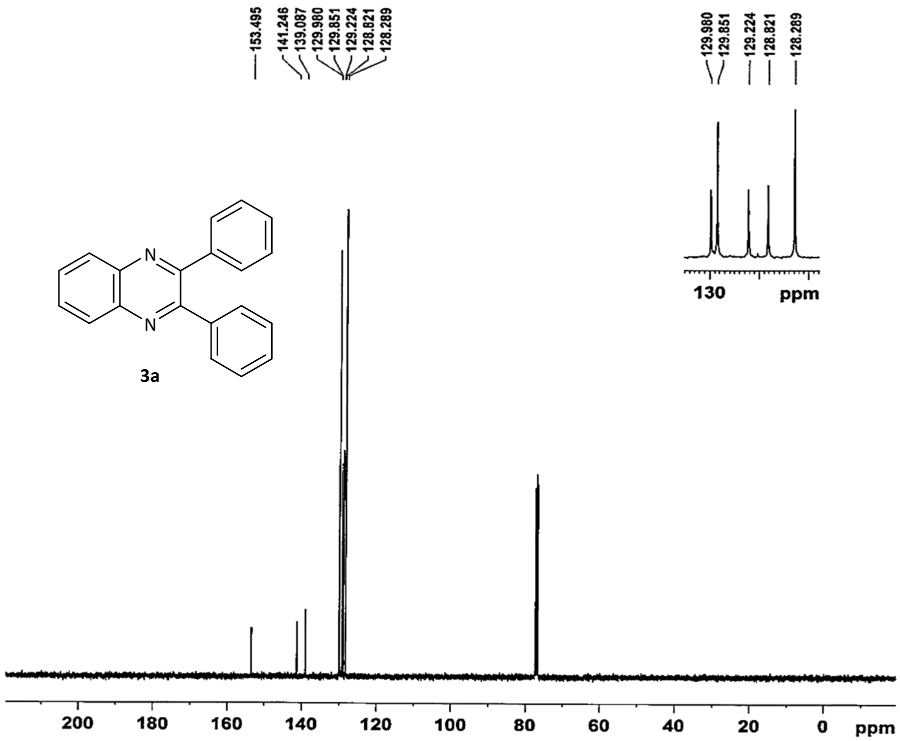

^{13}C NMR spectrum o of 2, 3-Bis-(4-bromo-phenyl)-quinoxaline (3b)

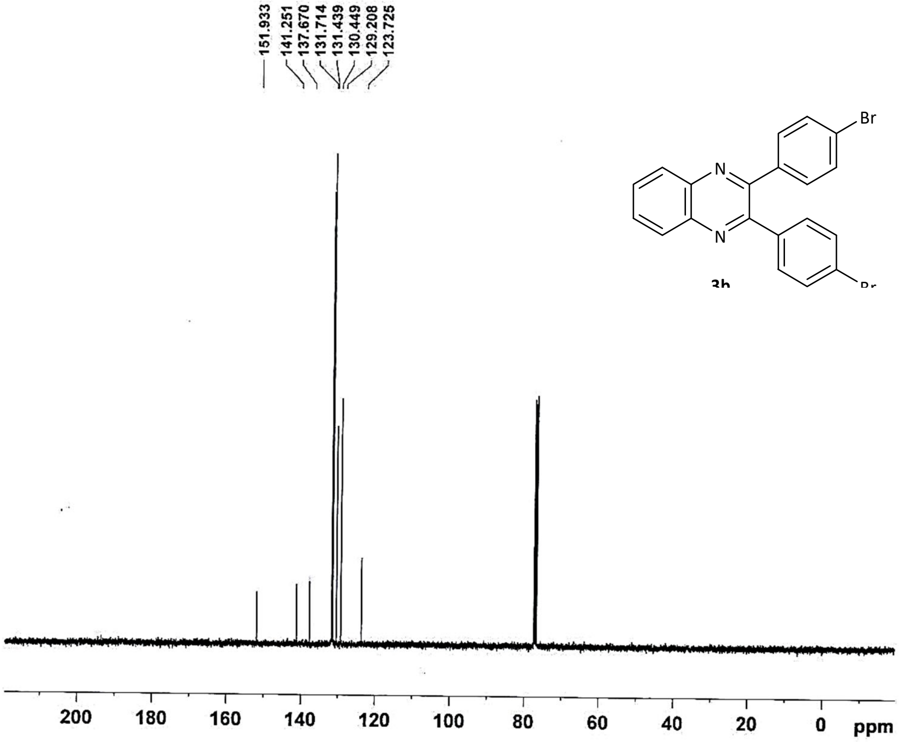

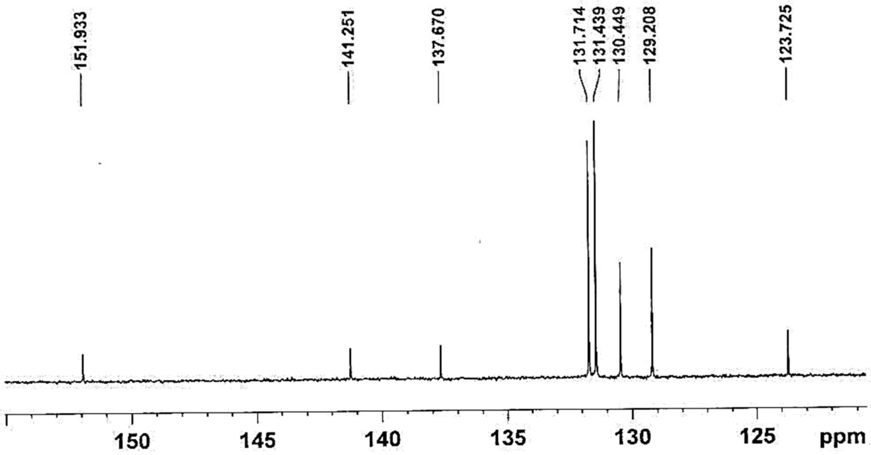

^{13}C NMR spectrum of 6-Methyl-2, 3-diphenyl-quinoxaline (3c)

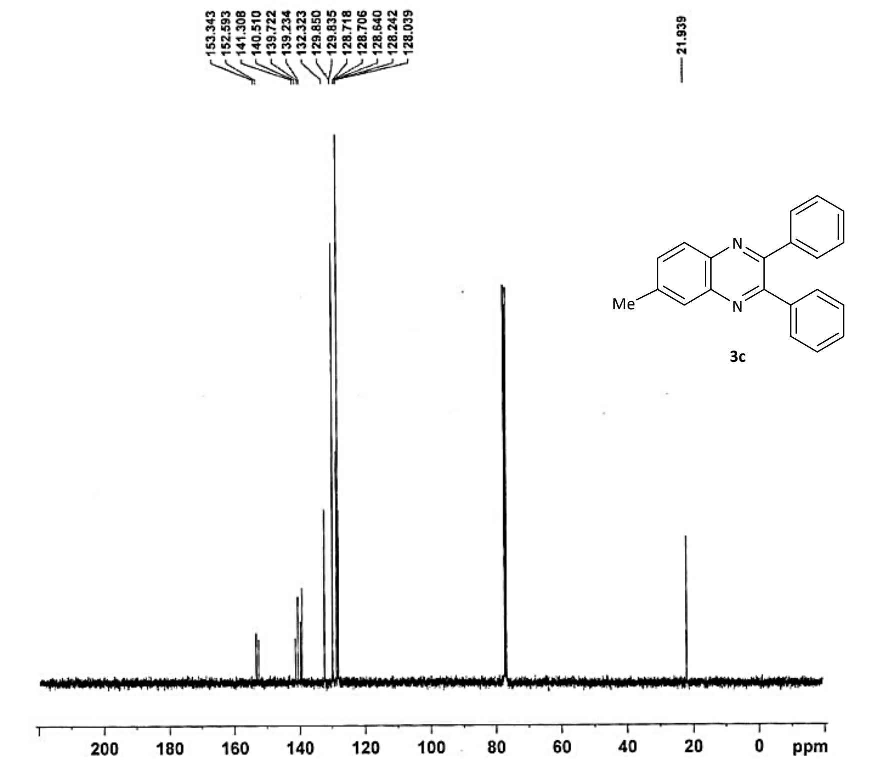

^{13}C NMR spectrum of 6-Methoxy-2,3-diphenyl-quinoxaline (3d)

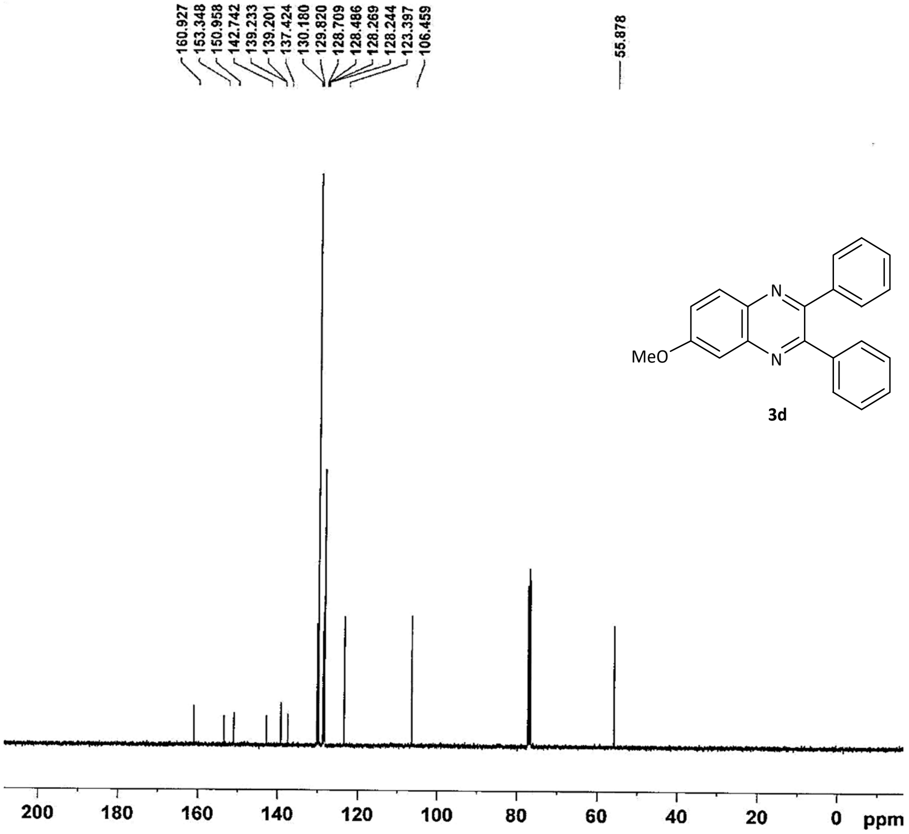

^{13}C NMR spectrum of 2, 3-Bis-(4-methoxy-phenyl)-6-nitro-quinoxaline (3e)

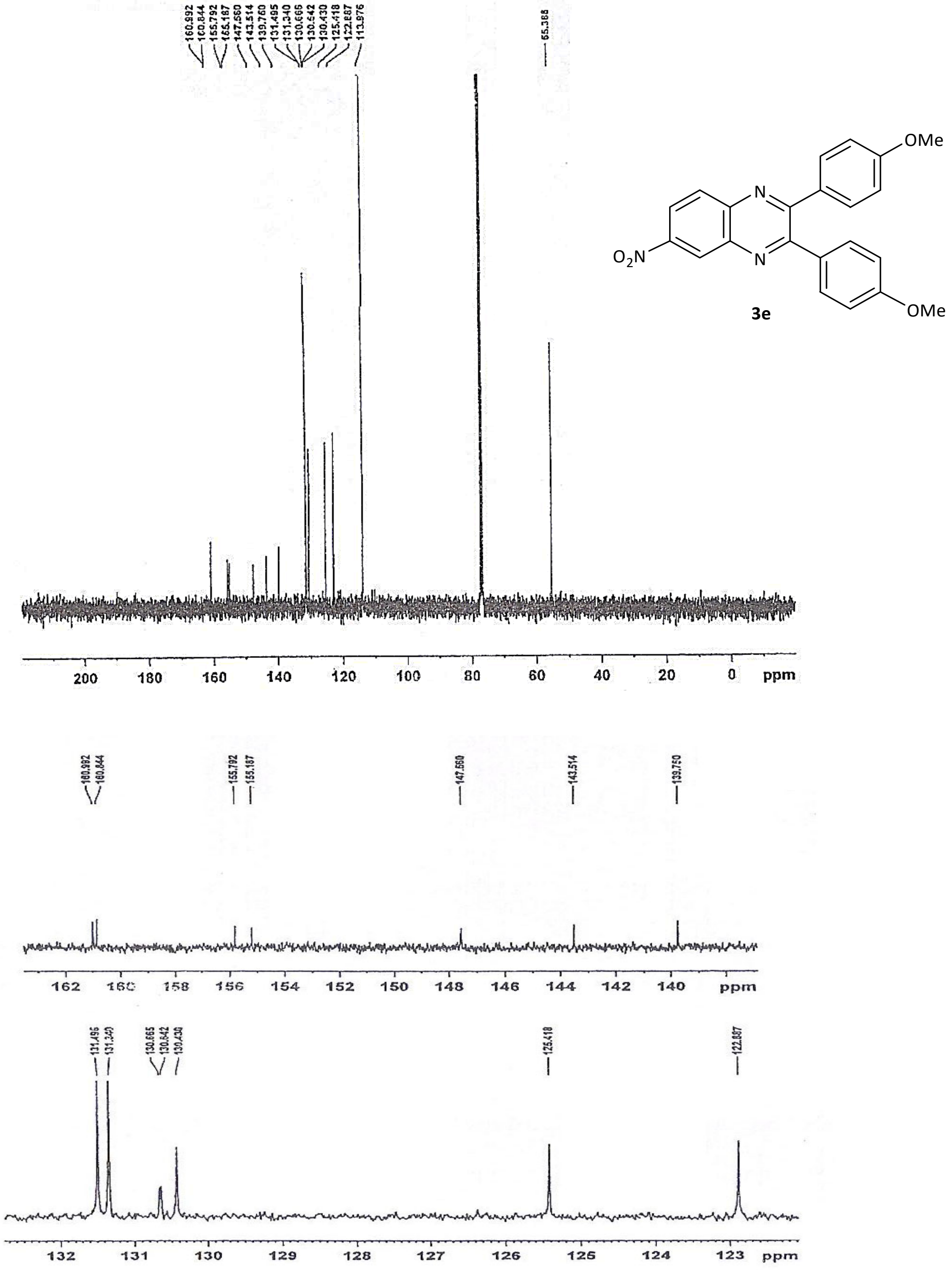

^{13}C NMR spectrum of 2, 3-bis-(4-methoxy-phenyl)-1, 4-diaza-benzo [a] antracene-7, 12-dione (3f)

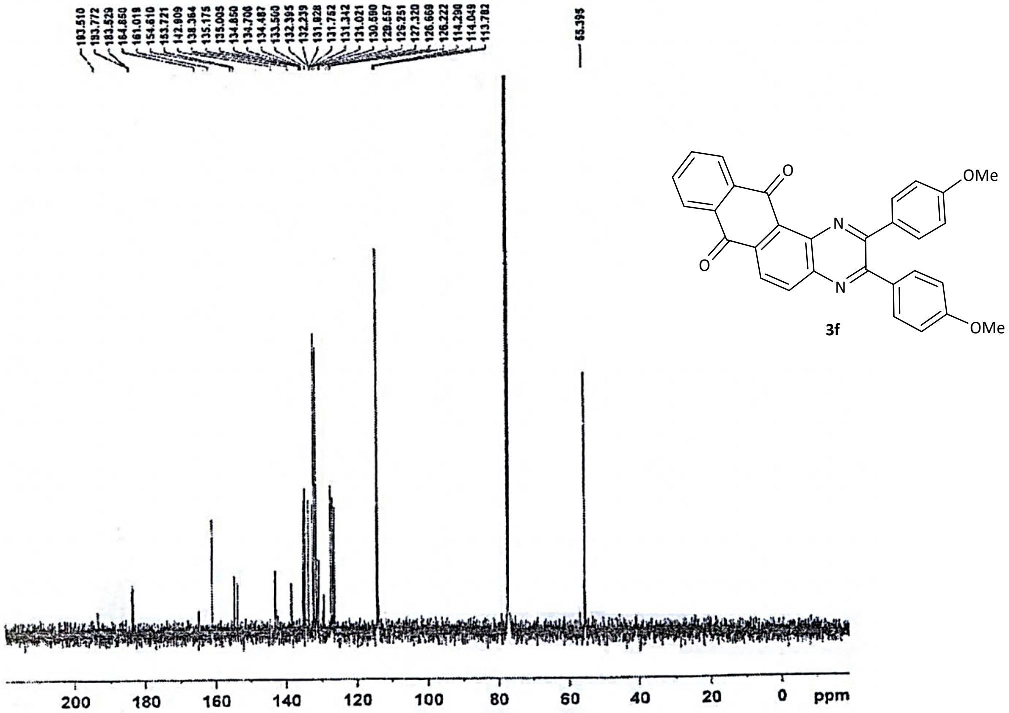

3f

^{13}C NMR spectrum of 11, 12 -dimethyl Dibenzo [a,c] phenazine (3g)

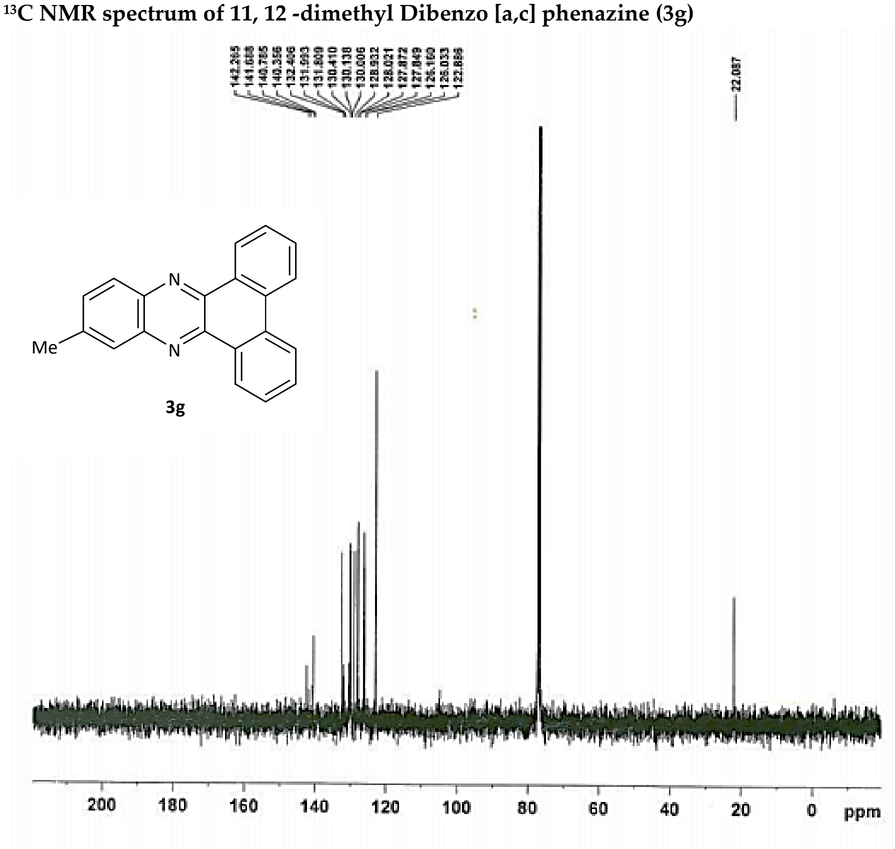

^{13}C NMR spectrum of 11, 12 -dimethyl Dibenzo [a, c] phenazine (3h)

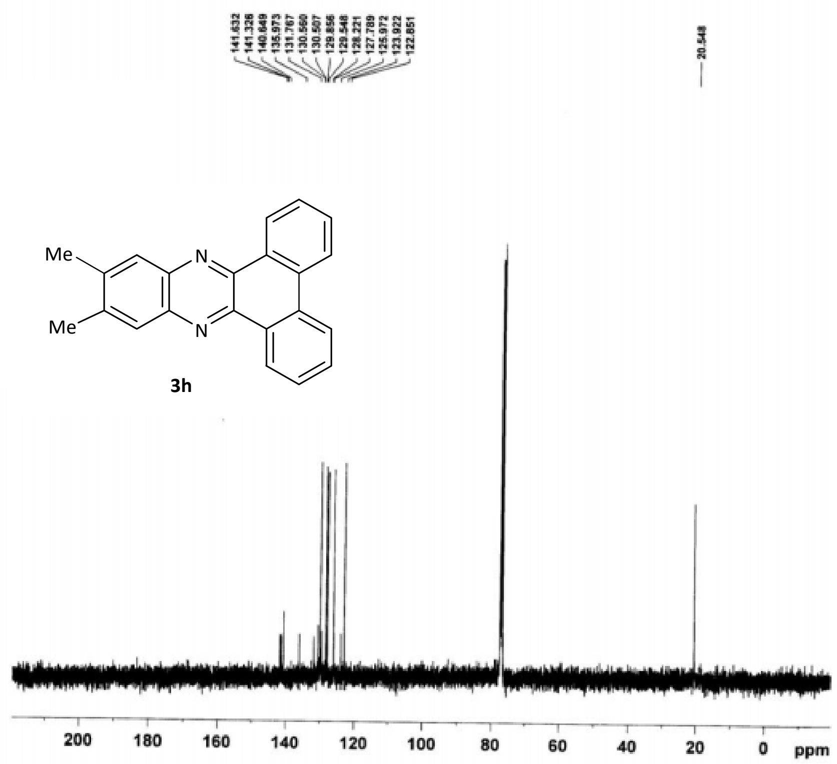

^{13}C NMR spectrum of Dibenzo [a, c] phenazine (3i):

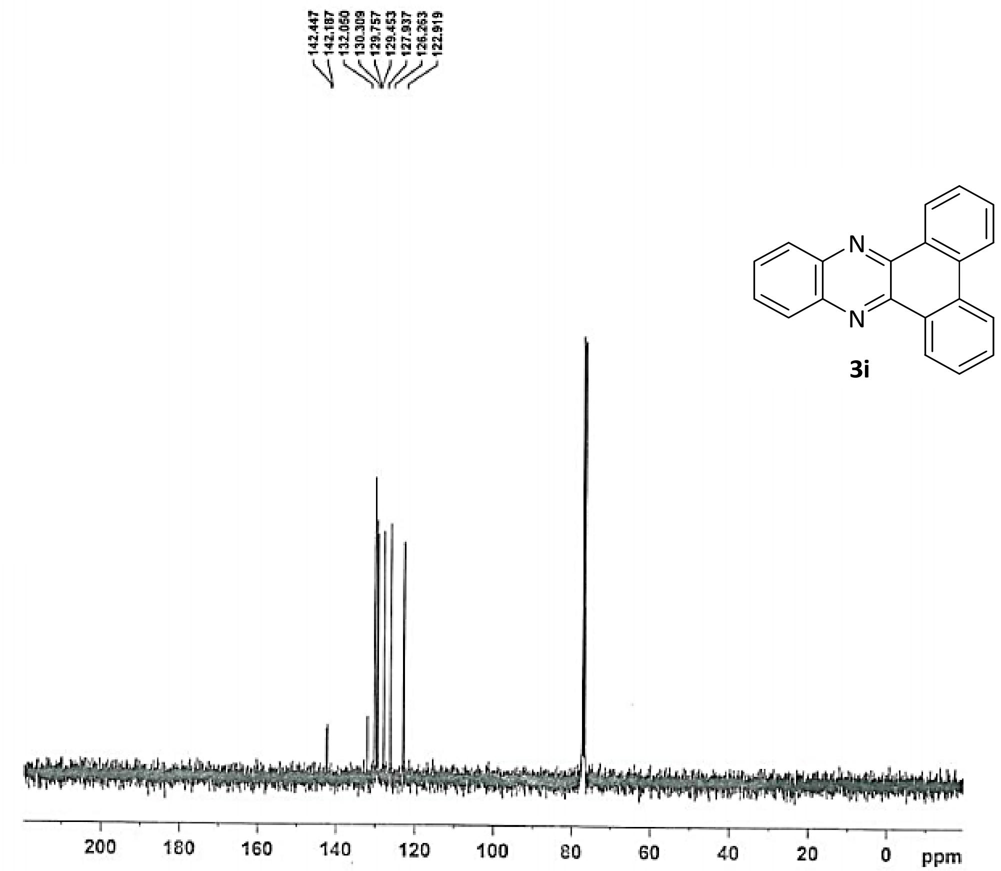

^{13}C NMR spectrum of Acenaphtho [1, 2-b] quinoxaline (3j)

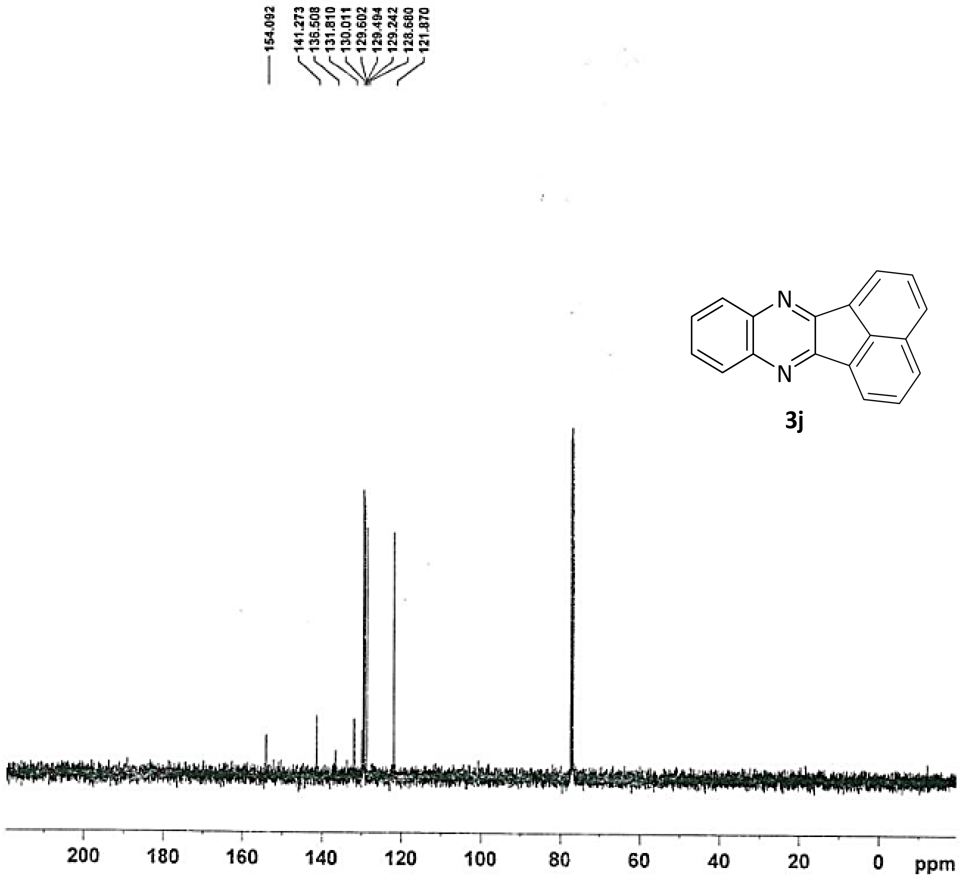

3j

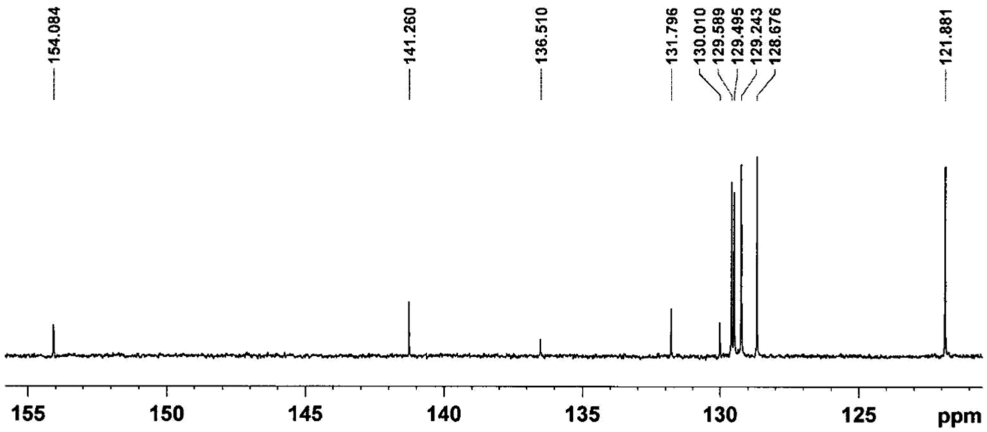

^{13}C NMR spectrum of 6-Methyl acenaphtho [1, 2-b] quinoxaline (3k)

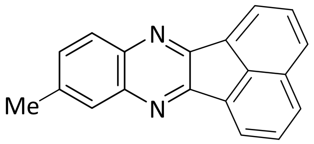

3k

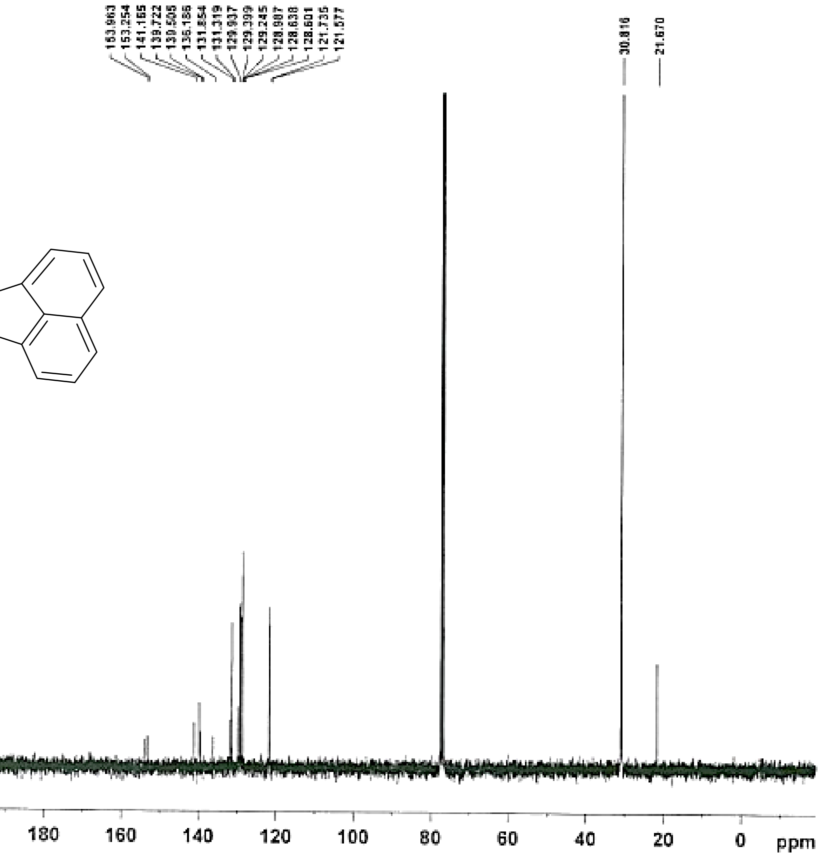

^{13}C NMR spectrum of 2, 3-Bis-(4-methoxy-phenyl)-6-nitro-quinoxaline (3l)

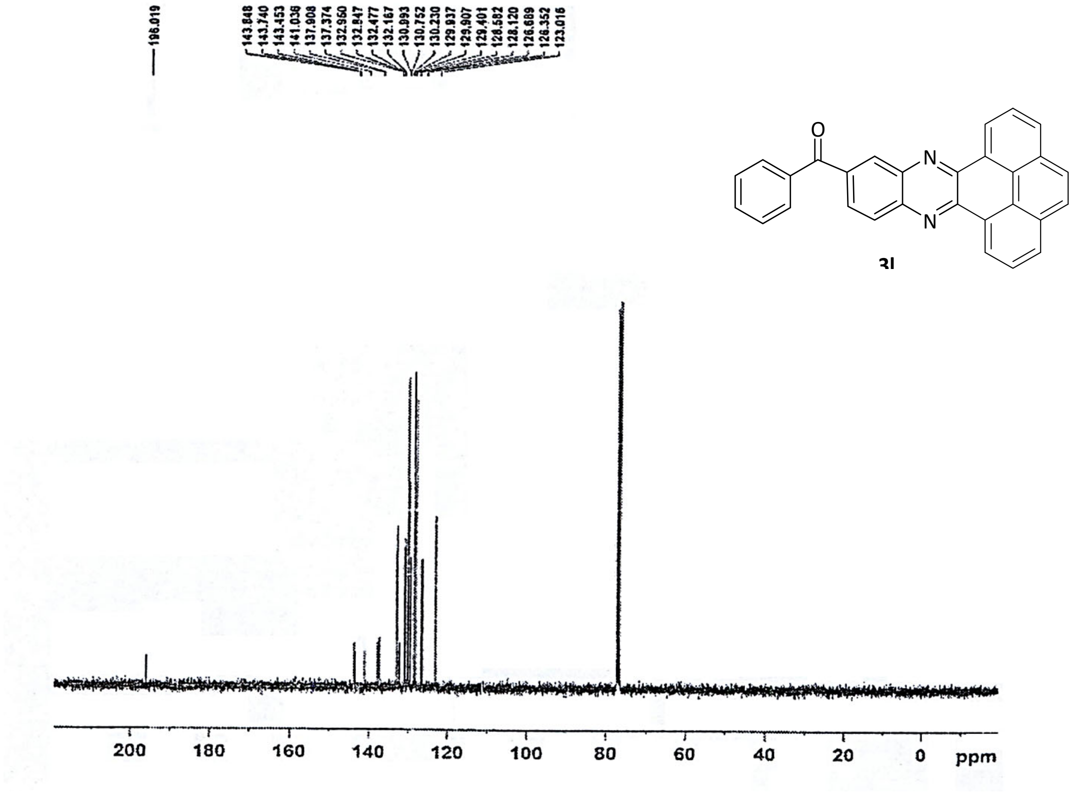

^{13}C NMR spectrum of 2, 3-Bis-(4-methoxy-phenyl)-6-nitro-quinoxaline (3m)

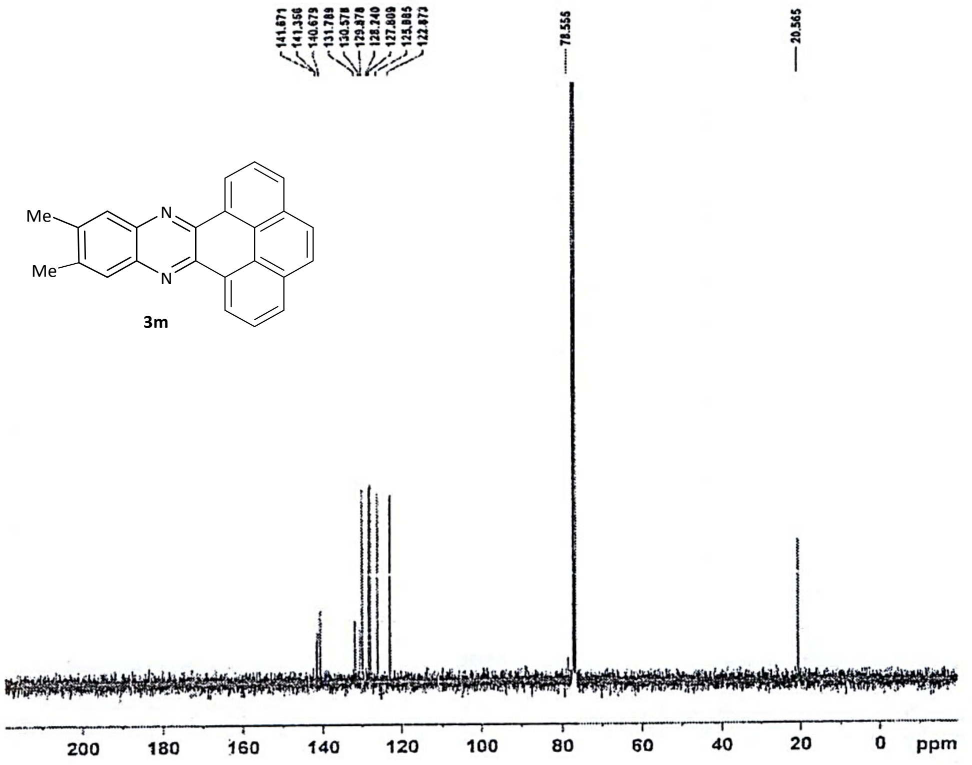

^{13}C NMR spectrum of Indeno [1, 2-b] quinoxalin-11-one (3n)

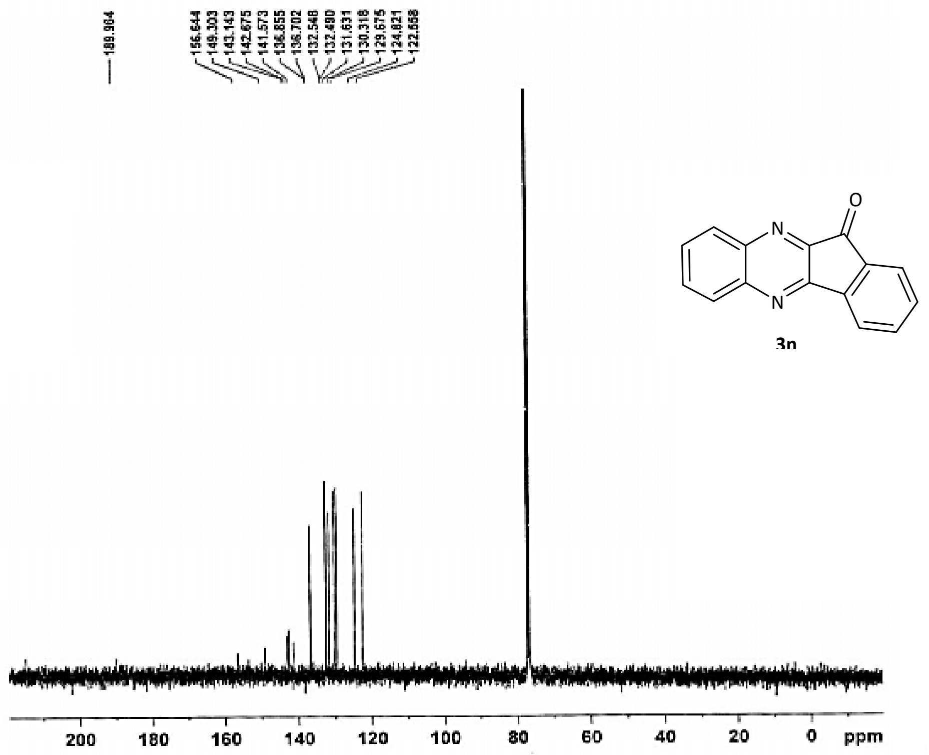

^{13}C NMR spectrum of 2, 3-Bis-(4-methoxy-phenyl)-6-nitro-quinoxaline (3o)

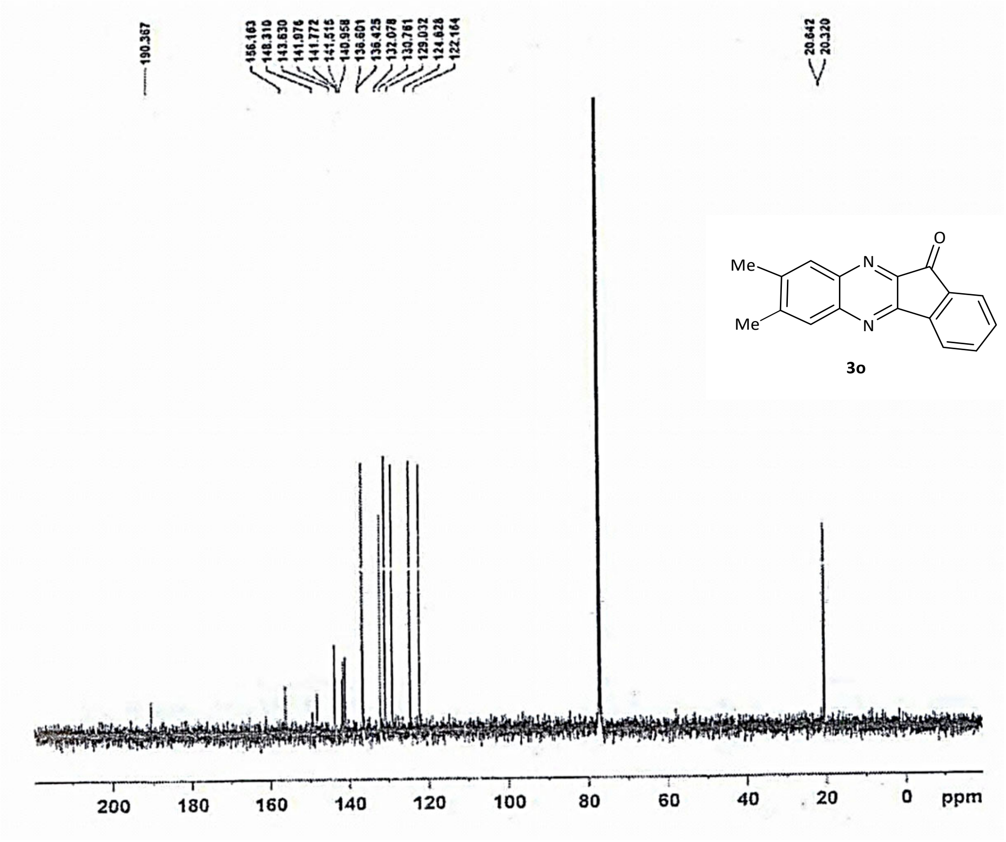

^{13}C NMR spectrum of 2, 3-Bis-(4-methoxy-phenyl)-6-nitro-quinoxaline (3p)

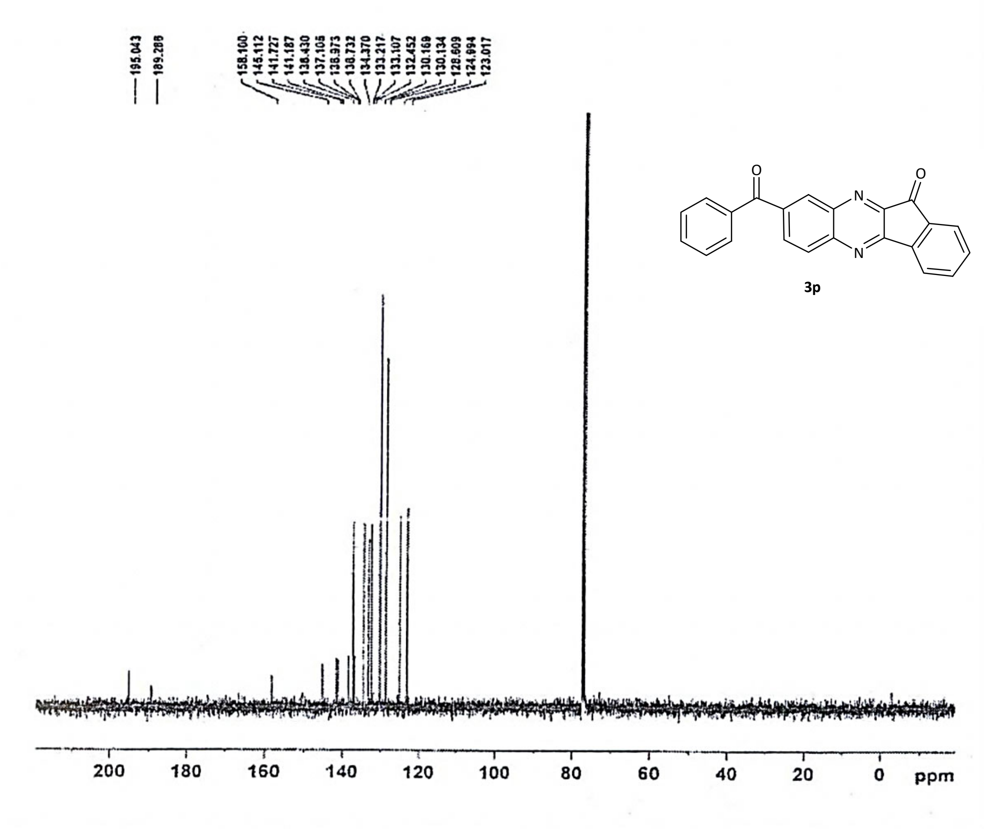

References

1. H. R. Ramananarivo, A. Solhy, J. Sebti, A. Smahi, M. Zahouily, J. Clark and S. Sebti, *ACS Sustain. Chem. Eng.*, 2013, **1**, 403-409.
2. R. Ebrahimi-Kahrizsangi, B. Nasiri-Tabrizi and A. Chami, *Particuology*, 2011, **9**, 537-544.
3. S. Music, N. Filipović-Vinceković and L. Sekovanić, *Brazil. J. Chem. Eng.* 2011, **28**, 89-94.
4. D. K. Bora, A. Braun, S. Erat, O. Safonova, T. Graule and E. C. Constable, *Curr. Appl. Phys.*, 2012, **12**, 817-825.
5. O. N. Karasyova, L. I. Ivanova, L. Z. Lakshtanov, L. Lövgren and S. Sjöberg, *Aqu. Geochem.*, 1998, **4**, 215-231.
6. M. Tajbakhsh, M. Bazzar, S. F. Ramzanian and M. Tajbakhsh, *Appl. Clay Sci.*, 2014, **88**, 178-185.
7. M. N. Sami Sajjadifar, Sara Miri, *Iran. J. Catal.* 2014, **4**, 55-61.
8. A. Go, G. Lee, J. Kim, S. Bae, B. M. Lee and B. H. Kim, *Tetrahedron*, 2015, **71**, 1215-1226.
9. P. K. Sahoo, C. Giri, T. S. Haldar, R. Puttreddy, K. Rissanen and P. Mal, *Eur. J. Org. Chem.*, 2016, **2016**, 1283-1291.
10. L. Carlier, M. Baron, A. Chamayou and G. Couarraze, *Tetrahedron Lett.*, 2011, **52**, 4686-4689.
11. F. Sadeghi B.; Karimi, *Iran. J. Catal.* 2013, **3**, 1 -7.

12. G. Kaupp, M. R. Naimi-Jamal and J. Schmeyers, *Chem. – A Eur. J.*, 2002, **8**, 594-600.
13. S. M. Baghbanian, *Chin. Chem. Lett.*, 2015, **26**, 1113-1116.
